# Dynamic and single cell characterization of a CRISPR-interference toolset in *Pseudomonas putida* KT2440 for β-ketoadipate production from *p*-coumarate^☆^

**DOI:** 10.1016/j.mec.2022.e00204

**Published:** 2022-08-28

**Authors:** Jacob A. Fenster, Allison Z. Werner, Jian Wei Tay, Matthew Gillen, Leo Schirokauer, Nicholas C. Hill, Audrey Watson, Kelsey J. Ramirez, Christopher W. Johnson, Gregg T. Beckham, Jeffrey C. Cameron, Carrie A. Eckert

**Affiliations:** a Department of Chemical and Biological Engineering, University of Colorado, Boulder, CO, 80309, USA; b Renewable and Sustainable Energy Institute, University of Colorado, Boulder, CO, 80309, USA; c Renewable Resources and Enabling Sciences Center, National Renewable Energy Laboratory, Golden, CO, 80401, USA; d BioFrontiers Institute, 3415 Colorado Avenue, Boulder, CO, 80309, USA; e Department of Biochemistry, University of Colorado, Boulder, CO, 80309, USA; f Department of Molecular and Cellular Biology, Harvard University, Cambridge, MA, 02138, USA; g National Bioenergy Center, National Renewable Energy Laboratory, Golden, CO 80401, USA; h Biosciences Division, Oak Ridge National Laboratory, Oak Ridge, TN, USA

**Keywords:** CRISPR interference, Single-cell analysis, Microbial lignin valorization, *Pseudomonas putida* KT2440, Heterogeneity

## Abstract

*Pseudomonas putida* KT2440 is a well-studied bacterium for the conversion of lignin-derived aromatic compounds to bioproducts. The development of advanced genetic tools in *P. putida* has reduced the turnaround time for hypothesis testing and enabled the construction of strains capable of producing various products of interest. Here, we evaluate an inducible CRISPR-interference (CRISPRi) toolset on fluorescent, essential, and metabolic targets. Nuclease-deficient Cas9 (dCas9) expressed with the arabinose (8K)-inducible promoter was shown to be tightly regulated across various media conditions and when targeting essential genes. In addition to bulk growth data, single cell time lapse microscopy was conducted, which revealed intrinsic heterogeneity in knockdown rate within an isoclonal population. The dynamics of knockdown were studied across genomic targets in exponentially-growing cells, revealing a universal 1.75 ± 0.38 h quiescent phase after induction where 1.5 ± 0.35 doublings occur before a phenotypic response is observed. To demonstrate application of this CRISPRi toolset, β-ketoadipate, a monomer for performance-advantaged nylon, was produced at a 4.39 ± 0.5 g/L and yield of 0.76 ± 0.10 mol/mol from *p*-coumarate, a hydroxycinnamic acid that can be derived from grasses. These cultivation metrics were achieved by using the higher strength IPTG (1K)-inducible promoter to knockdown the *pcaIJ* operon in the βKA pathway during early exponential phase. This allowed the majority of the carbon to be shunted into the desired product while eliminating the need for a supplemental carbon and energy source to support growth and maintenance.

## Highlights

• Developed an inducible dCas9-based CRISPR interference toolset in *Pseudomonas putida* KT2440.
• Characterized single-cell dynamics of fluorescent and essential gene knockdown.
• Applied the toolset for glucose-free production of β-ketoadipate from p-coumarate.
• Produced β-ketoadipate at titer of 4.39 ± 0.5 g/L and 0.76 ± 0.10 mol/mol yield.

## 1 Introduction

The genetic tractability of *Pseudomonas putida* KT2440 (hereafter *P. putida*), as well as its broad carbon metabolism and tolerance to diverse environmental and oxidative stressors, position this host as a promising chassis for biotechnological applications (Chavarría et al., 2013; Dos Santos et al., 2004; Nelson et al., 2002; Timmis, 2002)*.* Specifically, interest in using *P. putida* to convert lignin-related aromatic compounds to value-added products has grown due to the ability of this host to catabolize diverse aromatic substrates and tolerate cytotoxic compounds in lignin depolymerization streams (Abdelaziz et al., 2016; Becker and Wittmann, 2019; Beckham et al., 2016; Linger et al., 2014; Salvachúa et al., 2015; Weiland et al., 2022). In addition to its robustness in lignin rich environments, *P. putida* has a wealth of genetic tools that enable researchers to easily delete or introduce genes for the purpose of functional genomics and strain engineering, which was recently reviewed (Martin-Pascual et al., 2021). Natural products such as polyhydroxyalkanoates (Linger et al., 2014; Liu et al., 2017; Salvachúa et al., 2020), muconic acid (Johnson et al., 2019; Kohlstedt et al., 2018; Salvachua et al., 2018; Sonoki et al., 2018), lactate (Johnson and Beckham, 2015), pyruvate (Johnson and Beckham, 2015), β-ketoadipate (βKA) (Johnson et al., 2019; Okamura-Abe et al., 2016), 2-pyrone-4,6-dicarboxyilc acid (Lee et al., 2022), among others (Incha et al., 2020; Johnson et al., 2019; Okamura-Abe et al., 2016) have been produced in defined media from model lignin aromatics as well as from crude lignin liquor through traditional knock in-knock out methods. The general metabolic engineering strategy used in these examples in which the molecular targets are intermediates in catabolic pathways relies upon generating mutants with partial pathways that terminate at a desired product. Alternate carbon sources such as glucose or acetate are supplemented into the media to support growth and cell maintenance given that the majority of carbon is retained in the bioproduct. As an example, Johnson et al. 2019 produced 41.1 g/L βKA at near theoretical yield (1 mol/mol) from 4-hydroxybenzoate (4-10.13039/100014245HBA), although this *P. putida* Δ*pcaIJ* production strain required co-feeding glucose to support growth.

Interestingly, Sonoki et al., 2018 circumvented the need to feed additional carbon sources for the production of muconic acid in *Sphingobium* sp. SYK-6 and *P. putida*. These authors co-expressed enzymes in *P. putida* that forked metabolism at the common aromatic intermediate protocatechuate (PCA) into the product muconic acid while allowing some flux of PCA into the TCA cycle for growth and maintenance (Sonoki et al., 2018). This strategy was successful, resulting in 20% molar yield of muconic acid from a mixture of vanillate and 4-HBA. The same group used an alternate strategy where they engineered an isolated *Pseudomonas* strain that can grow on S, G, and H lignin to funnel G and H lignin into muconate while using the S lignin for growth; this strain produced 73% molar yield of muconate from vanillate while growing on 10 mM syringate (Shinoda et al., 2019).

As an alternative strategy to engineering bifurcated pathways, CRISPR interference (CRISPRi) can potentially be a viable approach to accumulate a desired product while allowing nominal flux through downstream pathways. This is accomplished by using a guide-RNA (gRNA) molecule to direct a catalytically dead Cas9 protein (dCas9) to a promoter or coding region of interest, allowing for titratable knockdown that can reduce gene expression from zero to 1000-fold versus baseline (Bikard et al., 2013; Mougiakos et al., 2018; Qi et al., 2013; Zhao et al., 2021). The bound dCas9+gRNA complex blocks the RNAP from initiating or elongating transcription, thereby knocking down gene expression (Bikard et al., 2013; Qi et al., 2013). Beyond modulating flux through downstream or alternate pathways, CRISPRi has been used for balancing flux through heterologous pathways (Kim et al., 2016; Tarasava et al., 2018), decoupling growth and production (Li et al., 2020), altering cell morphology for increased PHA accumulation (Elhadi et al., 2016), and downregulating transcriptional repressors for increased product flux (Cress et al., 2017; Kim et al., 2020). Many of these applications leverage the programmability and specificity of the gRNA to rapidly phenotype libraries of knockdown mutants, testing hundreds of thousands of strains in a single experiment (Todor et al., 2021; Fenster and Eckert, 2021).

Applications of CRISPRi rely upon a well characterized and tightly regulated expression system so that gene knockdown can be controlled and phenotypes can be accurately interpreted. Expression systems that are leaky result in background gene knockdown, preventing the construction of essential gene knockdown strains and the study of wild type -to-knockdown phenotype transition (Cui et al., 2018; Gordon et al., 2016; Tan et al., 2018). Furthermore, expression systems with over expressed dCas9 and/or gRNA suffer from general toxicity and off-target binding (Cui et al., 2018; Nielsen and Voigt, 2014). Therefore, robust tool design and characterization is paramount to realizing the benefits of CRISPRi experimentation. Towards this end, multiple groups have developed robust CRISPRi toolsets in *P. putida*, as reviewed recently (Martin-Pascual et al., 2021). These studies validated CRISPRi tools on fluorescent proteins (Batianis et al., 2020; Gauttam et al., 2021; Kim et al., 2020; Sun et al., 2018), essential genes such as *ftsZ* (Batianis et al., 2020; Gauttam et al., 2021; Tan et al., 2018), on multiple targets simultaneously (Banerjee et al., 2020; Batianis et al., 2020; Tan et al., 2018), for the production of indigoidine via growth based product coupling (Banerjee et al., 2020), and mevalonate via upregulation of glycerol utilization (Kim et al., 2020). Some of these toolsets are tightly regulated (Batianis et al., 2020; Gauttam et al., 2021) using the XylS/Pm regulator/promoter system or a combination of the P_*lac*_ and P_*bad*_ promoters for a tight off-state and high dynamic range, while others do not measure dynamic range (Banerjee et al., 2020; Kim et al., 2020; Sun et al., 2018) or have leaky off-states (Tan et al., 2018).

This work adds to the existing base of CRISPRi tools in *P. putida* by systematically characterizing additional CRISPRi toolsets on a bulk and single cell level against fluorescent, essential, and metabolic gene targets to measure the response time after induction as well as the knockdown heterogeneity in an isoclonal population. Last, we apply the system to produce βKA — a precursor for performance-advantaged nylon (Johnson et al., 2019; Rorrer et al., 2022) — from *p*-coumarate (*p-*CA) without an additional carbon source. In shake flasks, we produced βKA from *p-*CA at 4.4 g/L and 76% molar yield by knocking down *pcaIJ,* demonstrating a cultivation strategy for producing this value-added product in *P. putida* without addition of supplemental sugars.

## 2 Materials and methods

### 2.1 Plasmid construction

PCR was conducted using New England BioLabs Inc. (NEB) Q5® High-fidelity DNA Polymerase 2x Master Mix using standard protocols. Vectors were assembled using the NEBuilder® HiFi DNA Assembly Kit. Assembled vectors were electroporated into either *E. coli* EPI400 (Lucigen®), *E. coli* 10G (Lucigen®). Colonies were isolated on LB agar with appropriate antibiotics and inoculated into LB with appropriate antibiotic for plasmid extraction. Plasmids were extracted using the QIAprep® Spin Miniprep Kit (QIAGEN®) and whole plasmids or PCR amplicons were Sanger sequenced using GeneWiz®. Detailed plasmid construction information is located in the **Supplementary Material**.

### 2.2 Strain construction

Wild type *Pseudomonas putida* KT2440 ATCC® 47054 was used as the parent strain. dCas9 expression vectors were integrated between the hypothetical genes PP_4785 and PP_4786 using the *kan*/*sacB* suicide vector pK18sB (Jayakody et al., 2018) using an adapted method from Choi et al. 2006 andJohnson and Beckham, 2015 for transformation and integration, respectively. sfGFP expression vectors were integrated between the hypothetical genes PP_2984 and PP_2985 with Cas9/λ-Red mediated recombination via pCAS-RK2K using the pSEVA-gRNAT plasmid using the gRNA spacer sequence 5′-GCGGTTGCTCCACGCGCACA-3’ (Sun et al., 2018). Knockdown strains were constructed by electroporating gRNA expression vectors into dCas9 expression strains using previously described protocols. Plasmids were selected on LB 50 μg/mL kanamycin sulfate (kan50) plates at 30 °C overnight. Single colonies were inoculated into 5 mL LB kan50 in culture tubes and incubated at 30 °C, 200 RPM overnight and frozen in 15% (v/v) glycerol. Detailed strain construction information is provided in the **Supplementary Material and Methods**.

### 2.3 Culture conditions

*P. putida* was cultured in LB (Teknova: 1% tryptone, 0.5% yeast extract, 1% NaCl), modified M9 (6.78 g/L Na_2_HPO_4_, 3 g/L KH_2_PO_4_, 0.5 g/L NaCl, 1 g/L NH_4_Cl, 2 mM MgSO_4_, 100 μM CaCl_2_, 18 μM FeSO_4_) supplemented with 30 mM glucose or 20–40 mM *p*-coumaric acid, or MOPS (Neidhardt et al., 1974) (40 mM MOPS, 4 mM tricine, 50 mM NaCl, 1.32 mM K_2_HPO_4_, 9.52 mM NH_4_Cl, 0.523 mM MgCl_2_, 0.276 mM K_2_SO_4_, 0.01 mM FeSO_4_, 5x10^−4^ mM CaCl_2_, + micronutrient stock: 3x10^−6^ mM (NH_4_)_6_(MO_7_)_24_, 4x10^−4^ mM H_3_BO_3_, 3x10^−5^ mM CoCl_2_, 10^−5^ mM CuSO_4_, 8x10^−5^ mM MnCl_2_, 10^−5^ mM ZnSO_4_, adjusted to pH 7.2 with KOH) supplemented with 30 mM glucose or 20–40 mM *p*-coumaric acid as sole carbon source. Knockdown strains were grown in their corresponding media with 50 μg/mL kanamycin sulfate at 30 °C, 200 RPM. *p*-coumaric acid (*p*-CA) stocks, Sigma-Aldrich®, were made fresh the day of the experiment. *p*-CA was added to a stirred beaker with 80% of the final volume of ddH_2_O. The pH was adjusted to 7.8–8.35 with 1 M NaOH, until all visible particulate dissolved using sonication. The solution volume was adjusted to its final volume and the solution was filter sterilized with 0.2 μm cellulose membrane.

### 2.4 gRNA spacer design and construction

Spacer sequences were chosen to be specific to their target and to have a high predicted activity. Spacers were designed to be 20 nucleotides in length, and have a high on-target scores (>50) determined through the Benchling CRISPR design tool (www.benchling.com) that uses the algorithm published by Doench et al. (2016). Off-target effects were predicted using Cas-OFFinder with the *P. putida* KT2440 target genome (Bae et al., 2014). Spacers that had off-target binding sites with four or fewer mismatches were discarded. The non-targeting (NT) spacer sequence was created with a random DNA generator using 50% GC content and passed through the Cas-OFFinder software to assure it had no fewer than five mismatches with any potential genomic spacer. In addition, the non-targeting gRNA was experimentally validated to have the same transformation efficiency, as measured by CFU/μg plasmid DNA, as its parent vector, pBBR1MCS-2, when transformed into a cell harboring pCas9 which expresses active Cas9 (Cook et al., 2018) (data not shown). In the same experiment guides designed to target the chromosome were transformed resulting in 10^−5^ fewer CFU/μg plasmid DNA (data not shown). Spacer sequences were ordered as sense and anti-sense oligos, stitched together using PCR, and Gibson assembled into a PCR linearized pBB1MCS-2 gRNA expression backbone. This gRNA expression backbone contained a modified *tac* promoter which had the lac operator, *lacO*, truncated and placed the transcription start site at the first nucleotide of the gRNA spacer sequence (Elmore et al., 2017). For more details on gRNA cloning see the **Supplementary Materials and Methods.**

### 2.5 Plate reader experiments

**Fig. 1:**
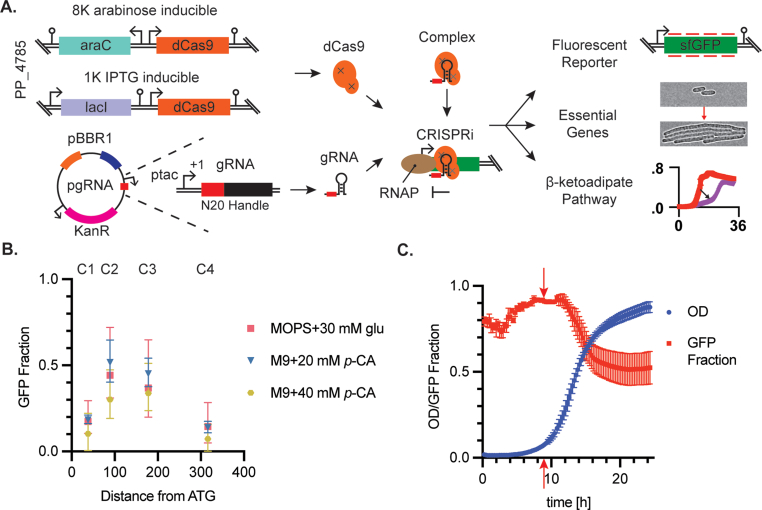
A CRISPRi toolset in P. putida. (**A**) A CRISPR-interference toolset was built in *P. putida* KT2440 by integrating the arabinose (8K) or IPTG (1K)-inducible dCas9 expression cassette into the neutral site at the PP_4785/PP_4786 intergenic locus. To target a gene of interest, a pgRNA vector with the pBBR1 origin of replication (blue) and the pBBR1 mobilizable elements, *mob* and *oriT* (orange), constitutively expresses a gRNA with the strong tac promoter was transformed into the dCas9 integrated strain and selected with kanamycin. Upon induction, dCas9 was expressed and complexed with the gRNA to knock down a gene of interest. This toolset was characterized on fluorescent reporters, essential genes, and *pcaIJ* within the βΚΑ pathway. (**B**) Steady state GFP fraction for the 8K inducible promoter B is plotted as a function of gRNA target location for 3 media conditions with saturating inducer molecule (1% (w/v) arabinose). gRNAs are named gGFP-C1, C2, C3, and C4 (labeled above axis); all four gRNAs target the *sfGFP* coding strand. Error bars represent 95% confidence intervals of three biological replicates. GFP fraction was calculated as the background subtracted experimental GFP concentration normalized by the background subtracted gNT GFP strain, see materials and methods section 2.6 for a detailed explanation. (**C**) sJF8KdCas9F + gGFP-C1 grown in MOPS +30 mM glucose were induced with 1% (w/v) arabinose at 10 h (red arrow) when the OD_600_ (blue line) was ∼0.1. Cellular GFP fraction was plotted as a function of time (red line) and shows a GFP decrease after induction over 5 h until stationary phase was reached. Error bars represent the standard deviation of biological triplicates. (For interpretation of the references to colour in this figure legend, the reader is referred to the Web version of this article.)

Steady state sfGFP concentration experiments (Fig. 1B, S2-S10) were conducted by pinning 96-well glycerol stocks of knockdown strains into 200 μL experimental media conditions tissue culture treated, Greiner μClear®, flat bottom, black-walled plates with lids containing condensation rings. Each knockdown strain media condition pair was studied in biological triplicate. The plate was loaded into a BioTek® Synergy™ 2 Microplate reader and programmed to incubate at 30 °C, shake quickly and continuously, and take OD and fluorescence measurements every 15 min. OD was measured at 600 nm and fluorescence was measured from the bottom of the plate at excitation/emission wavelength of 485/20,528/20 respectively directly after OD_600_ measurements. See **Supplementary Material** for a detailed description on how 96-well freezer stocks were constructed.

Dynamic CRISPRi experiments (Figs. 1C, 4B, S1 & S18) were conducted by inoculating knockdown strain glycerol stocks into LB kan50 and growing for 8–10 h at 30 °C, 200 RPM. These cultures were then diluted to an OD_600_ of 0.0001 in 20 mL of LB + kan in 125 mL shake flasks and allowed to grow for 12 h. These cultures were diluted to an OD_600_ of 0.05 in 20 mL LB kan50 and allowed to grow for 2–3 h until they reached early exponential growth, OD_600_ of 0.2–0.3. 10 mL of the cells were pelleted by centrifugation at 4,100×*g* for 10 min at room temperature. The supernatant was decanted and the cells were resuspended in the same volume of 1x MOPS or 1x M9 salts. The cells were pelleted again and resuspended in 500 μL (1/20^th^ of original volume) of 1x MOPS or 1x M9 salts and the OD_600_ of the inoculum was measured to be between 3 and 5. The inoculum was added to the experimental media to an initial OD_600_ of 0.1. Finally, 180 μL of the inoculated experimental media was dispensed into a 96-well, tissue culture treated, Greiner μClear®, flat bottom, black-walled plate with lids containing condensation rings in technical triplicate. Plates were grown as above. 20 μL of inducer solution, or sterile ddH_2_O, was added during early exponential growth by taking the plates out of the plate reader for 3–8 min *ftsZ* knockdown growth curves (Figs. 3A and S16A) Were conducted by inoculating knockdown freezer stocks into 5 mL LB kan50 and growing overnight 30 °C, 200 RPM. 1 μL overnight culture was added to 200 μL LB kan50 with or without 1% (w/v) arabinose in clear 96-well plates and grown as above.

### 2.6 GFP fraction calculation and growth rate analysis

For steady state GFP fraction experiments GFP concentrations were calculated at a given OD_600_ threshold based upon when the strains maintained steady state growth rates and cellular GFP concentrations. The threshold was set to OD_600_ = 0.1 for LB and 0.05 for all minimal media conditions. The fluorescence and OD_600_ was recorded at the threshold and the cellular GFP concentration was calculated by dividing the fluorescence signal by the OD_600_. Background fluorescence was subtracted via similar measurements of the corresponding non-fluorescent non-targeting dCas9 strain. The fraction GFP concentration was then calculated by dividing the GFP concentration in the knockdown strain by the GFP concentration in the positive GFP control strain. 95% bounds of the fraction GFP concentration were estimated by bootstrapping the distribution of cellular GFP concentrations in the knockdown and control strains and calculating the fraction GFP concentration for each pair of bootstrapped values repeated 10,000 times. Upper and lower values which bound 95% of the bootstrapped dataset were set as error limits. Limits less than zero were forced to zero as negative concentrations are not biologically possible and result from error comparing the knockdown strain to the non-fluorescent control.

Growth rates were calculated by finding the slope of the fitted a line to the natural log of OD_600_ as a function of time using three points either at a given OD_600_ threshold or at the time specified in the legend.

### 2.7 Single cell time lapse microscopy and slide preparation

Imaging was conducted according to (Hill et al., 2020). Brightfield and fluorescence images were taken using a commercial inverted wide-field microscope with a near-infrared–based Perfect Focus System (Nikon TiE). The sample was enclosed within an environmental chamber (ThermoFisher Lexan) which was temperature-controlled (Okolab). A high-speed light source with custom filter sets was used for imaging (Spectra X Light Engine, Lumencor, Beaverton, OR), along with a hardware-triggered and synchronized shutter for control of imaging. NIS Elements AR software (version 5.11.00 64-bits) with Jobs acquisition upgrade was used to control the microscope. Image acquisition was performed using a digital sCMOS camera (Hamamatsu ORCA-Flash4.0 V2+) with a 100 × oil immersion objective (1.45 numerical aperture; Nikon CF160 Plan Apochromat Lambda).

**Fig. 2:**
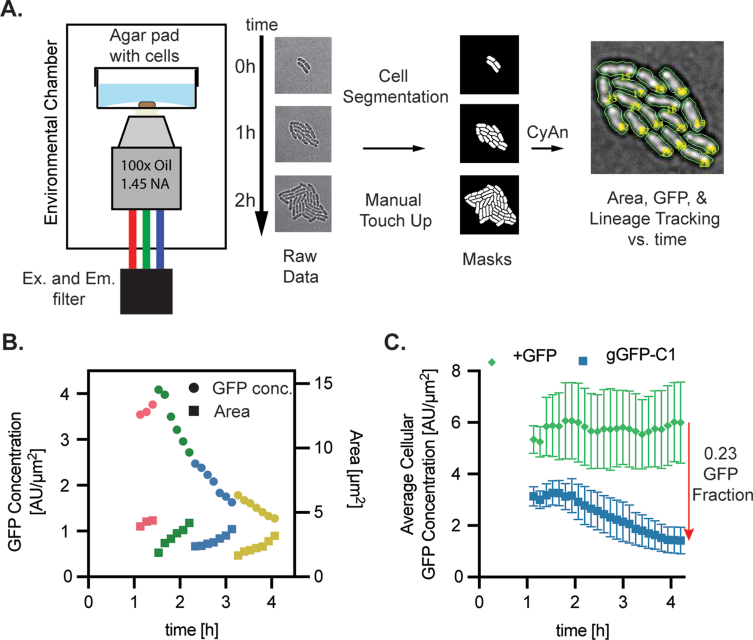
Single-cell dynamics of GFP knockdown in sJF8KdCas9F + gGFP-C1. (**A**) Single cell time lapse microscopy was conducted by spotting cells on an agar pad and inverting on a glass slide to confine the cells in 2-D. The slide was placed on the scope in an incubated chamber and images were taken every 8 min with brightfield, GFP channel, and brightfield-offset channels. Binary masks were generated by segmenting the raw data using a custom program, CyAn. This software also tracks cell lineages and extracts data such as area and GFP intensity to calculate GFP concentrations growth rates of each cell across its lifespan. (**B**) A lineage of one sJF8KdCas9F + gGFP-C1 with one descendent shown after each doubling. GFP concentration, circles, and cell area, squares, was plotted through each doubling. Pink is generation 3, green is generation 4, blue is generation 5, yellow is generation 6. (**C**) The GFP concentration of each cell for each frame was averaged to give the average cellular GFP concentration for sJF8KdCas9F + gNT (+GFP, green) and sJF8KdCas9F + gGFP-C1 (gGFP-C1, blue). Error bars represent the standard deviation of the individual cellular GFP concentration at each frame and therefore represent the intrinsic heterogeneity in the population of an isogenic culture. (For interpretation of the references to colour in this figure legend, the reader is referred to the Web version of this article.)

**Fig. 3:**
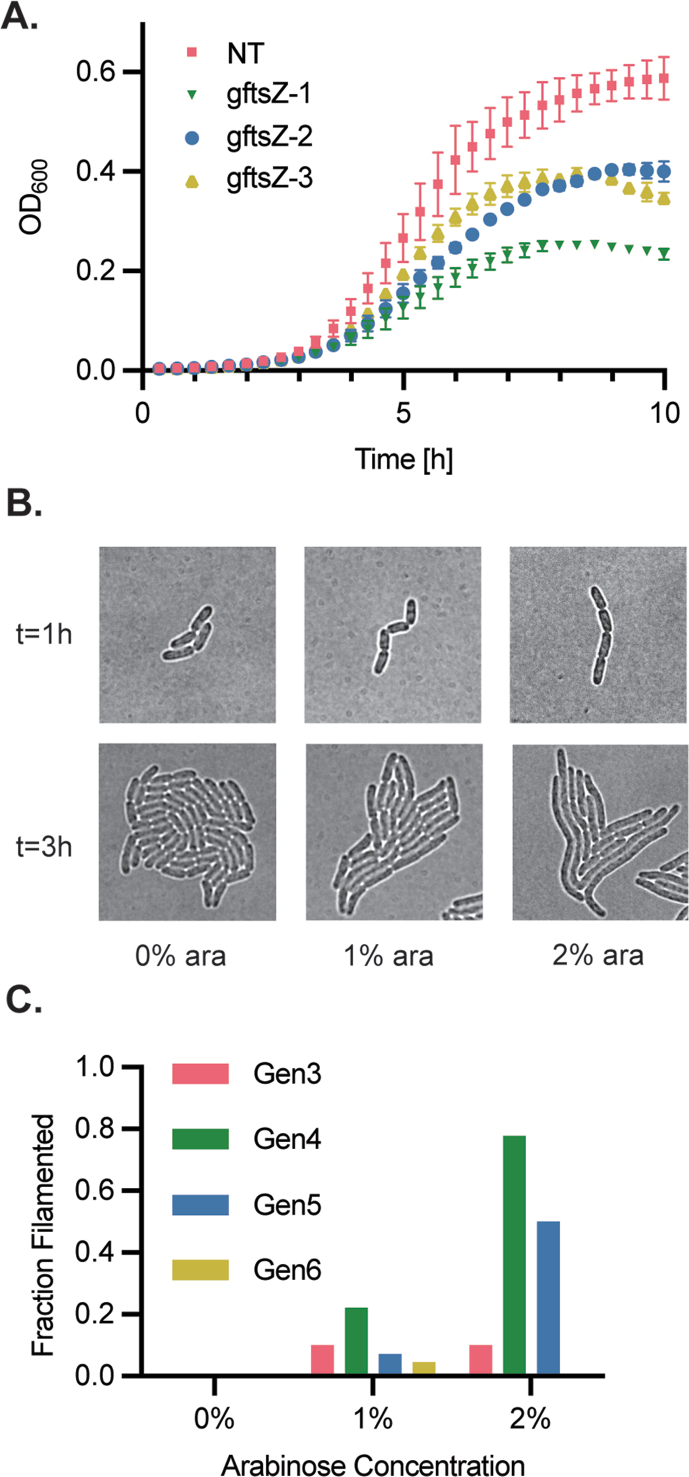
CRISPRi knockdown of ftsZ. (**A**) Growth of sJF8KdCas9+g*ftsZ*-1, -2, and -3 in LB + Kan_50_ with 1% (w/v) arabinose versus sJF8KdCas9+gNT in 96-well plates. Error bars represent the standard deviation of three biological replicates. (**B**) Images of *ftsZ* knockdown strains in 0%, 1%, and 2% (w/v) arabinose. sJF8KdCas9+g*ftsZ*-2 cells were spotted onto LB + Kan_50_ agarose plates with various arabinose concentrations. Filming commenced 1 h (2 doublings) after placing the slide in the incubated scope. Top images are at start of movie and bottom images are 2 h later. (**C**) Percentage of filamenting cells from each generation of an *ftsZ* knockdown cell at 0%, 1%, and 2% (w/v) arabinose percentage on LB agar. Filamenting cell were defined as a cell which never divides across the course of the experiment and grows larger than the largest cell in a sJF8KdCas9+gNT (WT phenotype) condition, here 9 μm^2^ cross sectional area (Fig. S15). The fraction of filamenting cells was measured and sorted based upon which generation gave rise to the phenotype. No error bars are shown because the plotted fractions represent all observations from three individual cells within a single biological replicate (n=12 for gen 3, n = 96 for gen 6).

Slides were prepared by spotting exponentially growing cells onto 1.5% w/v agar pads containing the various media conditions described below. Once cell spots dried for 15 min, agar pads were inverted and placed onto 4 well glass-bottomed slides (Ibidi μ-slide). The slide was then placed into the incubation chamber at 30 °C on the scope and allowed to reach thermal equilibrium for 45 min. At this point, separate x-y locations on the agar pad were chosen for filming. Movies commenced 1 h after incubation, with frames captured every 7–8 min. Knockdown strain pre-cultures were grown overnight from freezer stock in 5 mL LB kan in 14 mL culture tubes. These were diluted 100-fold into identical 5 mL cultures and allowed to reach early exponential phase, OD_600_ = 0.1–0.4. For Fig. 2B, 2**C**, and S11, cells were washed once with 1x M9 salts and normalized to an OD_600_=0.1 and 1 μL of this inoculum was spotted on a M9 + 30 mM glucose +1% arabinose kan50 agar pads and allowed to dry for 15 min. For Fig. 3B, 3C, and S16, cells were normalized to an OD_600_ of 0.1 in LB and spotted onto LB kan50 agar pads with 0, 1% and 2% arabinose. For Fig. S21, exponentially growing cells were prepared and washed as in section 2.10, diluted to OD_600_ of 0.1 in 1x MOPS salts then spotted on MOPS +40 mM p-CA + Kan50 + 0.25 mM IPTG agar pads.

### 2.8 Single cell segmentation

To identify individual cells in each frame (segmentation), a previously developed toolbox CyAn (Tay and Cameron, 2020) was run in MATLAB (R2020a) with custom segmentation scripts. Brightfield images were captured with a 2 μm focal plane offset to increase the contrast between the cells and the background and used for subsequent cell segmentation. First, cells were identified by using an intensity threshold. The intensity distribution of the image was fit to a Gaussian distribution and the mean of the Gaussian plus 1–2 times its standard deviation was used as a threshold. An initial binary mask was then generated by thresholding the offset brightfield images. This initial mask was then filtered by removing objects below 200 and above 50,000 pixels in size. The remaining masks were then grown by 6 pixels to accurately represent cell size. This protocol left artifacts, fused cells, and some omitted cells. Thus, a final mask was then generated by manual correction in Fiji.

### 2.9 Cell tracking and analysis

CyAn (Tay and Cameron, 2020) was used to implement a version of Jaqaman's tracking algorithm (Jaqaman et al., 2008) to link data from a single cell between frames. Each tracked cell was assigned a unique cell ID, and properties such as its area and GFP intensity were recorded at each frame of the movie. CyAn also recognized cell divisions and recorded related mother-daughter cell IDs and generation. Additional properties were calculated from the tracked data: The growth rate for each cell was calculated by finding the slope of the fitted line to natural log of the cell length over time for a cells lifespan. Cellular average GFP intensity was calculated by dividing the total GFP intensity by the cell area. The change in cellular GFP concentration over time, dF/dt, was calculated by finding the slope of the fitted line to the cellular GFP concentration versus time data over a cells lifespan.

### 2.10 Shake flask experiments

The inoculum for flask scale-up experiments was made as the inoculum for dynamic CRISPRi experiments as described above, except scaled up 10-fold. Early exponentially growing sJF1KdCas9+g*pcaIJ*-2 cells (OD_600_=0.2–0.3) were washed 1x with MOPS salts and resuspended in MOPS salts at 1/20^th^ of the original volume. This inoculum was added to 50 mL MOPS Kan50 + 40 mM *p*-CA with or without 0.25 mM IPTG to a starting OD_600_=0.1 and grown at 30 °C, 200 RPM. 0.5 mL of 25 mM IPTG was added to the exponential phase induced condition at 14 h when the OD_600_ increased to 0.12.

### 2.11 Quantification of metabolites

βKA was analyzed and quantified as levulinic acid after acid hydrolysis was performed as previously described (Rorrer et al., 2022).

Aromatic acids *p*-CA acid, 4-HBA, and PCA were analyzed by ultra-high pressure liquid chromatography with diode array detection as detailed previously (Kuatsjah et al., 2022) with the extension of the linear quantitation range to be 1 μg/mL-1,000 μg/mL for all analytes.

## 3 Results

### 3.1 Building a tunable CRISPR-interference toolset in P. putida KT2440

We sought to build *P. putida* strains with inducible, chromosomally-integrated dCas9 expression cassettes and plasmid-encoded, constitutively-expressed gRNAs targeting either a fluorescent reporter, an essential gene, or *pcaIJ* to induce βKA accumulation (Fig. 1A). For dCas9 expression, two inducible promoters were selected: the 1K-J23107 IPTG-inducible promoter and the 8K arabinose-inducible promoter (Cook et al., 2018). The catalytically inactive *S. pyogenes* Cas9 (D10A, H841A, dCas9) was cloned into an integration vector (Jinek et al., 2012) and integrated into the *P. putida* genome between the hypothetical genes PP_4785 and PP_4786, generating IPTG (1K)- or arabinose (8K)-inducible dCas9 strains sJF1KdCas9 and sJF8KdCas9, respectively (Table 1). Integrating dCas9 did not alter fitness versus the wild type strain in minimal glucose media (Fig. S1).

**Table 1:** Select strains used in this work. See *Supplemental Information* for full list and corresponding construction details.

Strain name	Genotype (Description)	Source
*P. putida* KT2440	Wild-type (WT) *Pseudomonas putida* KT2440	ATCC® 47054
sJF1KdCas9	*P. putida* KT2440 PP_4785/PP_4786::P_1K_:*dCas9* (IPTG-inducible dCas9 expression)	This work
sJF8KdCas9	*P. putida* KT2440 PP_4785/PP_4786::P_8K_:*dCas9* (arabinose-inducible dCas9 expression)	This work
sJF1KdCas9F	*P. putida* KT2440 PP_4785/PP_4786::P_1K_:*dCas9* PP_2984:P_*tac*_:*sfGFP* (IPTG-inducible dCas9 expression, constitutive sfGFP expression)	This work
sJF8KdCas9F	*P. putida* KT2440 PP_4785/PP_4786::P_8K_:*dCas9* PP_2984:P_*tac*_:*sfGFP* (arabinose-inducible dCas9 expression, constitutive sfGFP expression)	This work
sJF1KdCas9+gNT	Non-targeting control, IPTG inducible, non-fluorescent	This work
sJF8KdCas9+gNT	Non-targeting control, arabinose inducible, non-fluorescent	This work
sJF1KdCas9F + gNT	+GFP non-targeting control, IPTG inducible	This work
sJF8KdCas9F + gNT	+GFP non-targeting control, arabinose inducible	This work
sJF8KdCas9F + gGFP-C1	sJF8KdCas9F transformed with coding strand targeting pJFgRNA_GFP-C1	This work
sJF8KdCas9F + g*ftsZ*-2	sJF8KdCas9F transformed with coding strand targeting pJFgRNA_*ftsZ*-2	This work
sJF1KdCas9+g*pcaIJ*-2	*P. putida* KT2440 PP_4785/PP_4786::P_1K_:*dCas9* + pJFgRNA_pcaIJ-2 (IPTG-inducible dCas9 expression + constitutive plasmid-based expression of gRNA targeting the coding strand of *pcaIJ*)	This work

Targeting gRNAs and a non-targeting (NT) gRNA control were expressed from a strong constitutive *tac* promoter (Elmore et al., 2017) from the pBBR1MCS-2 backbone with a kanamycin resistance marker. The NT control vector, pJFgRNA_NT, will hereafter be referred to as “gNT”.

### *3.2* *CRISPRi-mediated knockdown of* sfGFP

To assess the inducibility of dCas9-mediated knock-down, we first targeted a genome-integrated fluorescent reporter. Expression of a *P. putida* codon optimized *sfGFP* cassette, see Supplemental Materials and Methods, was driven by a derivative of the strong and constitutive P_*tac*_ (Elmore et al., 2017) and integrated between the hypothetical genes PP_2984 and PP_2985. The 1K and 8K dCas9 systems (Cook et al., 2018) were respectively integrated into this GFP expression strain to construct the fluorescent strains sJF1KdCas9F and sJF8KdCas9F (Table 1). Eight targeting gRNAs were designed to target the first 300 bp after the start codon of *sfGFP,* either binding to the coding strand (gGFP-C1, gGFP-C2, gGFP-C3, gGFP-C4) or the template strand (gGFP-T1, gGFP-T2, gGFP-T3, gGFP-T4) (Table 2). These gRNA expression vectors were transformed into sJF1KdCas9F and sJF8KdCas9F to construct *sfGFP* knockdown strains (Table 1). The steady state sfGFP concentration of each strain was calculated during exponential growth by dividing the sfGFP signal by the OD_600_ at a given threshold OD_600_ when the GFP/OD signal remained constant over time. Background fluorescence was then subtracted from the sfGFP concentration. To compare the relative knockdown between growth conditions with varying expression levels of sfGFP in the positive control, the sfGFP fraction was calculated by normalizing the cellular sfGFP concentration of the knockdown strain by the non-targeting fluorescent control strain. See materials and methods section 2.6 for more detail.

**Table 2:** Select plasmids used in this work. See *Supplemental Information* for full list and corresponding construction details.

Plasmid name	Short name	Description
pJFgRNA-NT	gNT	Non-targeting gRNA Ptac expression vector with pBBR1MCS-2 ORI
pJFgRNA_GFP-C1	gGFP-C1	P_*tac*_ expression of gRNA that targets *sfGFP* at P13 (38 bp), coding strand
pJFgRNA_GFP-C2	gGFP-C2	P_*tac*_ expression of gRNA that targets *sfGFP* at R30 (89 bp), coding strand
pJFgRNA_GFP-C3	gGFP-C3	P_*tac*_ expression of gRNA that targets *sfGFP* at L60 (178 bp), coding strand
pJFgRNA_GFP-C4	gGFP-C4	P_*tac*_ expression of gRNA that targets *sfGFP* at Y106 (316 bp), coding strand
pJFgRNA_*ftsZ*-2	g*ftsZ*-2	P_*tac*_ expression of gRNA that targets *ftsZ* at P12 (36 bp), coding strand
pJFgRNA_*pcaIJ*-2	g*pcaIJ*-2	P_*tac*_ expression of gRNA that targets *pcaI* at A63 (190 bp), coding strand

To evaluate the dynamic range and robustness of knockdown, *sfGFP* strains were grown in four media conditions supplemented with three inducer concentrations. The sJF1KdCas9F strains were induced with 0, 0.025, and 0.25 mM IPTG and the sJF8KdCas9F strains were induced with 0, 0.1, and 1% (w/v) arabinose (Fig. S2& S7). Error was propagated by bootstrapping the measured distribution of knockdown and setting limits that contained 95% of the resulting values. Any negative knockdown limits were forced to equal zero as negative values are not physically possible and result from noise in measuring background fluorescence (Materials and Methods).

The promoter driving dCas9 expression had a large effect on the dynamic range of knockdown. The 8K system showed a tightly regulated ‘off’ state in all media conditions and with all gRNA designs tested (Fig. S3). Upon adding 1% (w/v) arabinose to the 8K sfGFP knockdown strains, the sfGFP concentration reduced by 50–83% of the positive control (Fig. 1B). The 1K system reduced GFP close to baseline even without IPTG induction, indicating that dCas9 expression was leaky from this promoter (Figure S2, S4 & S5). This leaky dCas9 expression is consistent with previous CRISPRi studies in *P. putida* (Cook et al., 2018; Gauttam et al., 2021; Tan et al., 2018). The 8K system was titratable across the media conditions tested, except for the MOPS 30 mM glucose condition in which the 0.1% (w/v) arabinose condition did not differ significantly from the uninduced condition, (Fig. S3). The 1K system was only titratable for the guide RNA gGFP-C3 in specific media conditions, (Fig. S5). The dynamic range of the 8K promoter was higher due to the high baseline knockdown rate in the 1K system (Figs. S2–S4). While the gRNA design (Fig. S6), dCas9 promoter (Fig. S2), and induction concentration (Figs. S2, S3, & S5) affected the GFP knockdown, the media condition did not (Figs. 1B and S4).

Previous studies developing CRISPRi design rules have reported that template strand binding gRNAs either have negligible effect on gene expression or illicit a small level of knockdown (Bikard et al., 2013; Cui et al., 2018; Qi et al., 2013). All four gRNAs targeting the template strand of *sfGFP* did not reduce the cellular sfGFP concentration (Fig. S7)**.** While gRNAs gGFP-T3 and -T4 did not affect the sfGFP concentration versus the gNT control, surprisingly, strains carrying gGFP-T1 and gGFP-T2 exhibited a relative increase in cellular level of sfGFP during exponential growth compared to the non-targeting control (Fig. S7). The increase in sfGFP concentration for gGFP-T1 and gGFP-T2 with the 8K system occurred at the highest induction level, while the increase was seen at all three IPTG concentrations for the 1K system, additionally increasing with inducer concentration, suggesting that the resulting increase is inducible (Fig. S7). No consistent growth rate defects were seen with the template strand targeting gRNAs suggesting that this could be a gRNA sequence specific phenomenon (Figs. S9–S10).

Next, the dynamics of sfGFP knockdown were studied. The strain sJF8KdCas9F + gGFP-C1 was chosen for further characterization because the 8K arabinose-inducible system was titratable and gGFP-C1 was a high-performing gRNA for knockdown. sJF8KdCas9F + gGFP-C1 was grown in MOPS plus 30 mM glucose without arabinose and cultivated in 96-well plates until cultures reached early exponential phase (OD_600_ = 0.1), at which point the plate was removed and 1% (w/v) arabinose was added.

After 1.2 doublings (1 h 45 min) post-induction, the cellular GFP concentration began to decline (Fig. 1C). After a total of 2.5 doublings after induction, the average sfGFP concentration dropped to 54% of the gNT + GFP control strain (sJF8KdCas9F + gNT) (Fig. 1C). Presumably more than 2.5 doublings are required to reach the steady state sfGFP fraction of 0.17 that was measured in Fig. 1B.

### 3.3 Single-cell dynamics of the CRISPRi-mediated sfGFP knockdown

To gain an understanding of the heterogeneity of knockdown in an isoclonal population, the sJF8KdCas9F + gGFP-C1 was filmed with single cell time lapse microscopy and cell tracking was implemented to measure cellular sfGFP concentrations through mother and daughter cells after induction (Fig. 2A). Cells were spotted on M9 plus 30 mM glucose agar pads with 1% (w/v) arabinose, inverted onto glass slides, allowed to reach thermal equilibrium for 45 min, and then filming commenced at 1 h with images in the brightfield and GFP channels taken every 8 min. Three microcolonies were segmented from each condition. Initial colonies contained an average of four cells such that two doublings had occurred before the start of filming.

A single lineage of sJF8KdCas9F + gGFP-C1 is shown in Fig. 2B, where the sfGFP concentration and cell area are plotted over time for a single cell and one of its descendants through each of three doublings. The sfGFP concentration dropped over time while the growth was at steady state. The average cellular sfGFP concentration of all segmented cells per frame remains constant for the gNT control strain but declines with the C1 gRNA (Fig. 2C). Error bars represent the variation within the isoclonal population.

While the average sfGFP concentration of the sJF8kdCas9+gGFP-C1 knockdown cells decreases over time, individual lineages deviate from this trend (Fig. S11A). This was also seen for the positive control where very few lineages stay at a constant sfGFP concentration through growth, (Fig. S11A). Plotting the derivative of sfGFP concentration with time over an individual cell's lifespan, dF/dt, reveals this heterogeneity (Fig. S11B). The + sfGFP strain had both positive and negative dF/dt values within all generations, where the knockdown strain showed both positive and negative values for generation three, but these values became unanimously negative for the fourth and fifth generation (Fig. S11B). While there was variation in the growth rates for both the +sfGFP and +gGFP-C1 knockdown cells (Fig. S11C), this variation does not explain the heterogeneity in dF/dt (Fig. S11D). This observed knockdown heterogeneity is important for understanding bulk phenotypes as a distribution of knockdown levels rather than a constant value.

### 3.4 CRISPRi-mediated knockdown of essential genes

We next applied the toolset to study essential gene mutant phenotypes. Three essential genes were selected: *ftsZ,* required for cell division; *dnaA*, required for DNA replication; and *rpoD,* involved in protein expression. Three gRNAs were designed for each gene. No transformants were recovered after repeated attempts with sJF1KdCas9, again demonstrating the leakiness of the 1K promoter system. In contrast, essential gene knockdown strains constructed with sJF8KdCas9 showed inducible toxicity when plated with and without 1% (w/v) arabinose after transformation (Figs. S12–S14). Induced strains showed reduced colony number and size regardless of the mechanism of lethality (Figs. S12–S14). One guide (g*rpoD*-3) showed a less-than-lethal phenotype when induced (Fig. S13). The *ftsZ* knockdown strains were studied in further detail because the CRISPRi phenotypic response is well documented in *P. putida* KT2440 and because the morphological phenotype lends itself to microscopic observation (Batianis et al., 2020; Gauttam et al., 2021; Tan et al., 2018). Initial bulk experiments in LB revealed both growth rate and final OD_600_ defects caused by inducing *ftsZ* knockdown in sJF8KdCas9 (Figs. 3A and S16A).

To investigate this phenotype on a single cell level, sJF8KdCas9+g*ftsZ*-2 was studied with time lapse microscopy. Three microcolonies were segmented from generations 3–6, and the CyAn tookbox was used to track division events and cell area over time (Tay and Cameron, 2020). The filamenting phenotype threshold was determined by looking at the distribution of cell area of the non-targeting control strain and picking 9 μm^2^ as the threshold because it was larger than the largest measured control cell (Fig. S15). No filamenting cells were observed after 6 generations of 150 initial microcolonies for the sJF8KdCas9+g*ftsZ*-2 with 0% arabinose (data not shown). Increasing the arabinose concentration increased the percentage of filamented cells (Fig. 3B–C). Even though filamenting cells did not divide, growth rate was similar to sJF8KdCas9+gNT (Fig. S16B). Generation 4 produced the largest percentage of filamenting cells in both the 1% and 2% (w/v) arabinose conditions, demonstrating the need for *ftsZ* dilution before division is prevented. After 3 h of growth, filamenting cells made up 40% and 93% of total cell area on the 1% and 2% (w/v) arabinose conditions, respectively. There was no difference between total cell area over time between the mutant and knockdown lines or between induction concentrations for the course of the movie (Fig. S16C).

### *3.5* *CRISPRi-mediated production of β-ketoadipate from* p-*coumarate*

**Fig. 4:**
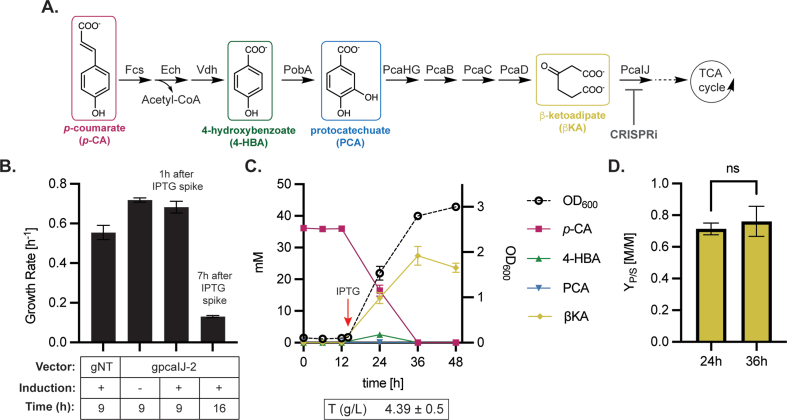
Controlling flux through the β-ketoadipate pathway. (**A**) *p-*CA is catabolized through the aromatic intermediates 4-HBA, PCA, and βKA, where PcaIJ converts βKA to β-ketoadipyl-CoA. βKA can be accumulated as product by deletion of *pcaIJ,* or further metabolized into a unit of succinate and acetyl-CoA which supports growth via the tricarboxylic acid (TCA) cycle. The overall reaction is *p*-CA + ATP + CoA + 2O_2_ + H_2_O -> βKA + acetyl-CoA + AMP + diphosphate + CO_2_ + 2H^+^ (**B**) Growth rate of *pcaIJ* knockdown strain induced during early exponential phase grown in 20 mM *p*-CA as the sole carbon source. The growth rates of the non-targeting control strain, gNT and the uninduced knockdown strain g*pcaIJ*-2 were calculated at 9h of growth during early exponential phase. The *pcaIJ* knockdown strain was induced with 0.25 mM IPTG at 8h and the growth rate was measured at 9 and 16h of growth. (**C**) 50 mL cultures containing 40 mM p-CA were inoculated at OD_600_ = 0.1 and induced with 0.25 mM IPTG 14 h later, red arrow. The concentration of *p*-CA, 4-HBA, PCA, and βKA, in the supernatant was plotted as a function of time alongside the OD_600_. Titer, T, was calculated at 36 h when *p*-CA dropped to zero (**D**) The product yield Y_P/S_ was plotted for the 24 h and 36 h time point relative to the initial substrate concentration. A paired *t*-test between the 24 h and 36 h time points showed no significant difference (p=0.29). All error bars represent the standard deviation of three biological replicates. (For interpretation of the references to colour in this figure legend, the reader is referred to the Web version of this article.)

Lastly, we sought to apply the CRISPRi toolset to produce βKA from the lignin-related aromatic compound *p-*CA without an additional carbon source. *p-*CA is catabolized by *P. putida* through βKA, where PcaIJ is a CoA-transferase that converts βKA to β-ketoadipyl-CoA, which is metabolized further to enter the TCA cycle (Fig. 4A). An acetyl-CoA is generated in the conversion of *p-*CA to PCA; thus, upon repression of *pcaIJ,* a limited amount of cellular growth on acetyl-CoA could occur even with accumulation of βKA. In our previous work, glucose was also added as an additional source of carbon and energy for growth (Johnson et al., 2019). We hypothesized that upon induction of CRISPRi-mediated *pcaIJ* repression, a growth defect would occur and βKA would accumulate, without the need for a supplementary carbon source. In addition, incomplete knockdown of *pcaIJ* could allow for some βKA to be catabolized into a succinate and acetyl-CoA. Another possible route for the growth of a *pcaIJ* knockdown strain is the acid-catalyzed decarboxylation of βKA to levulinic acid, which can be catabolized (Rand et al., 2017).

Two gRNAs, g*pcaIJ*-1 and g*pcaIJ*-2, were transformed into either arabinose or IPTG-inducible dCas9 backgrounds to generate four βKA production strains (Table 1). Each strain was spotted on minimal agar pads containing 20 mM *p-*CA as the sole carbon source, with or without inducer (1% (w/v) arabinose or 0.25 mM IPTG). The largest inducible growth defect was seen when inducing strain sJF1KdCas9+g*pcaIJ*-2 with 0.25 mM IPTG (Fig. S17B). Under this condition, the same number of CFUs were formed relative to the sJF1KdCas9+gNT strain, but the size of colonies in sJF1KdCas9+g*pcaIJ*-2 was reduced after 24 h of incubation (Fig. S17B). As expected, adding glucose to the IPTG-induced pad rescued growth from the *pcaIJ* knockdown strain (Fig. S17B).

We next evaluated growth on *p-*CA as the sole carbon source in 96-well plate cultivations. With 0.25 mM IPTG induction at 0 h, the *pcaIJ* knockdown strain sJF1KdCas9+g*pcaIJ*-2 grew with a 2.8-fold decrease in growth rate that was maintained until 24 h (Figs. S18A–B). The addition of glucose rescued the growth rate, indicating that the growth defect was due to decreased aromatic catabolism through βKA as opposed to general decreased fitness. While the growth rate was reduced throughout the cultivation, the knockdown strain reached a similar final OD_600_ as the control strain (Fig. S18A). The dynamics of knocking down *pcaIJ* was studied by inducing dCas9 expression during early exponential growth (OD_600_ = 0.05, at 9 h) (Fig. 4B, S18C-D). Two hours after induction (1.7 doublings), the growth rate began to decrease, and 7 h after induction (3.3 doublings), the growth rate slowed to 5.2-fold lower than its original value (Fig. S18D). Regardless of the induction condition, sJF1KdCas9+g*pcaIJ*-2 eventually grew to the same final OD_600_ as the control (Fig. S18D).

Single cell time lapse microscopy was used to study the dynamics inducing *pcaIJ* knockdown at time zero. sJF1KdCas9+g*pcaIJ*-2 was grown on a solid agar pad with 40 mM *p-*CA and 0.25 mM IPTG. It took four doublings for there to be a difference in growth rate between the *pcaIJ* knockdown strain and the non-targeting control (Fig. S21). Growth rate heterogeneity was observed in this isoclonal population (Fig. S21).

To measure the accumulation of βKA through *pcaIJ* knockdown, shake flask cultivations were performed by inoculating sJF1KdCas9+g*pcaIJ*-2 in 50 mL of MOPS minimal media plus 40 mM *p-*CA as the sole carbon source. IPTG was added to the cultures once after the lag phase (OD_600_=0.12, at 14 h). The concentration of *p-*CA, βKA, and two upstream aromatic intermediates, 4-HBA and PCA were quantified along the growth curve, (Fig. 4A, 4C). Inducing during the lag phase (0 h) resulted in poorer production relative to the early exponential induced cells (Fig. S20). The control strain sJF1KdCas9+gNT produced only small quantities of βKA with no βKA detected at 36 h (Fig. S19).

Cell growth was observed, along with conversion of *p-*CA to βKA with a final titer of 4.39 ± 0.5 g/L and yield of 0.76 ± 0.10 mol βKA per mol *p*-*CA* (Fig. 4C). The product yield (Y_P/S_) did not change over the course of the experiment (Fig. 4D), implying that the knockdown of *pcaIJ* remained constant albeit incomplete throughout the experiment. No upstream aromatic metabolites measured accumulated during the cultivation (Fig. 4C). In addition, no βKA was produced in the non-targeting control strains (Fig. S19). Inducing the culture at time zero rather than during early exponential growth resulted in lower βKA yields (Fig. S20) and a higher final OD_600_ value than the exponentially induced condition (Figs. 4C and S20).

## 4 Discussion

This work provides a well characterized CRISPRi toolset for *P. putida* that can be used for functional genomics as well as bioprocessing. The tightly regulated 8K arabinose inducible dCas9 system enabled the construction and study of essential gene knockdown strains. If higher induction is needed to study a phenotype of interest, the 1K system provided greater knockdown rates at the cost of a leaky off state. The arabinose system characterized here had a similar dynamic range as the Xyl/P_*m*_ promoter from Batianis et al. (2020), which responds to 3-methylbenzoate (3 MB). Arabinose has the advantage of being orthogonal to *P. putida* KT2440 metabolism, where 3 MB induces the expression of the BenABCDKZF benzoate ortho-cleavage route (Domínguez-Cuevas et al., 2006). Gauttam et al., 2020 achieved an even higher dynamic range than presented in this work by inducing dCas9 with P_*lac*_ and the sgRNA with P_*ara*_ promoter, allowing for the construction of essential gene knockdown strains like *ftsZ* with stronger growth defects than presented here. Beyond the toolset presented here, our characterization of CRISPRi dynamics on a bulk and single cell level as well as our demonstration of a successful proof of concept cultivation requiring dynamic knockdown provide insights that are applicable for future experimental designs and cultivation optimizations regardless of which specific CRISPRi toolset is chosen for strain construction.

By characterizing a set of eight guides targeting the template and coding strand of sfGFP, basic CRISPRi design rules were found to be mostly consistent with previous CRISPRi tool development studies in bacteria (Bikard et al., 2013; Gauttam et al., 2021; Qi et al., 2013). Here we show that targeting the coding strand is effective for reducing gene expression. In addition, we show that the sequence of the targeting region of the gRNA is more important than the distance from the start codon, as was seen by guides gGFP-C1 and gGFP-C4 outperforming gGFP-C3, (Figs. 1B and S6). This has also been reported by groups studying *E. coli* (Cui et al., 2018; Gauttam et al., 2021).

Here we have observed increased sfGFP expression by targeting the template strand with gGFP-T1 and gGFP-T2. Previous studies have described nominal knockdown when targeting the template strand of fluorescent and essential genes, but none led to increases in protein concentration (Bikard et al., 2013; Qi et al., 2013; Rousset et al., 2018). We did not attempt to identify the mechanism by which the GFP concentration in exponentially growing cells surpassed wild type by up to five times, but the result was gRNA design specific, inducible as well as reproducible across media conditions, biological replicates, and toolsets (Fig. S7).

For liquid culture exponentially growing cells, there was a 1.75 ± 0.38 h, or 1.5 ± 0.35 doubling quiescent phase after induction with arabinose or IPTG where constant protein levels are maintained. This was seen when knocking down both *sfGFP* and *pcaIJ* where the GFP concentration and growth rate, respectively, remained constant, (Fig. 1, Fig. 4B). After this quiescent phase the target protein diluted to a steady state value over multiple generations depending upon the target gene. This also is consistent with the *ftsZ* knockdown where two doublings were required to begin to see filamenting cells (Fig. 3C).

It took over six doublings to reduce sfGFP levels down to its steady state value, whereas the *pcaIJ* knockdown strain required only 3.3 doublings (Figs. 2C and S18D). While we do not know the steady state FtsZ protein level directly, knockdown strains reached a maximum filamenting phenotype after 3 doublings (Fig. 3C). More than three additional doublings were required for sfGFP equilibration, which can be partially explained by its long rate of degradation (Andersen et al., 1998; Pédelacq et al., 2006). Swapping the protease stable sfGFP used here with a less stable version would most likely result in fewer doublings required to achieve a steady state concentration after CRISPRi induction.

Higher CRISPRi knockdown was needed to elicit an effect when knocking down *pcaIJ* versus GFP and essential genes, as targeting of *pcaIJ* with the arabinose-inducible system resulted in no growth defects (Fig. S17). The higher level of knockdown needed to observe a growth defect could be due to the upregulation of *pcaIJ* through PcaR activation, which responds to increased βKA levels (Parales and Harwood, 1993). This result shows that generating a desired phenotypic response can be gene specific and require the fine tuning of CRISPRi induction levels.

A molar yield for βKA of 76 ± 10% was achieved in shake flask by feeding solely *p*-CA to a *P. putida pcaIJ* knockdown strain (Fig. 4C). While yields close to theoretical have been reported when feeding other engineered *P. putida* strains model lignin monomers, these studies have deleted *pcaIJ* and require the feeding of an additional carbon source for cell growth and maintenance (Johnson et al., 2019; Okamura-Abe et al., 2016). Here a fraction of the fed *p*-CA was used for cell growth while most of the carbon was shunted into a product. The induction scheme was critical for this type of CRISPRi-induced product accumulation. Inducing during early exponential increased the titer and yield versus inducing during lag phase. This is likely explained by a reduced knockdown of *pcaIJ* which would allow for more substrate to be converted into biomass or byproducts instead of βΚΑ. Indeed, an almost 2-fold difference in minimum growth rate was observed in plate reader experiments depending upon when inducer was added (Fig. S18B&D). This work provides a proof-of-concept glucose-free strategy to convert lignin monomers into value added products via dynamic CRIPSRi knockdown.

Heterogeneity in context of CRISPRi experiments is important to understand bulk phenomena such as phenotypic escape from essential gene knockdowns (Peters et al., 2016) as well as for a more complete understanding of measurements taken during pooled functional genomics screens (Boettcher et al., 2019). Single cell time lapse microscopy allowed us to quantify the heterogeneity that exists through lineages of an isoclonal population. Observing sfGFP knockdown, both the positive sfGFP control and the knockdown strains showed a large variation in expression levels that is not explained by variation in growth rate (Figs. 2C and S11). Furthermore, the sfGFP concentrations within +GFP cells overlapped with the sfGFP concentrations in some knockdown cells (Fig. S10A). This variation in gene reduction can explain escape phenotypes that do not require base changes in the CRISPRi machinery. For example, the *ftsZ* knockdown strain exhibited a binary filamenting phenotype where cells that maintained a high enough expression of *ftsZ* divided as wild type. The volume fraction of wild type-like cells within the *ftsZ* knockdown population after 3 h of growth decreased dramatically from 60% to 7% when increasing arabinose from 1% to 2% (w/v), showing that increasing induction can shift the distribution of cells that elicit the desired phenotype. Last, variation in single cell growth rate was observed in the *pcaIJ* knockdown strain. As growth rate and βKA production were inversely related (Fig. 4, S18-S20), this variation in growth rate likely corresponds to variation in single cell productivities. A possible explanation for why inducing during exponential growth produced higher titers of product and lower growth rates could be that lag phase induction enriched for a high growth rate and low producing population. Indeed, low producing subpopulations in isoclonal populations have been shown to lead to lower yields in bioprocessing (Mustafi et al., 2012; Rugbjerg et al., 2018; Xiao et al., 2016). A potential strategy for increasing yield beyond what is presented here could be to further engineer the dCas9 and gRNA expression machinery for reduced noise in knockdown (Bar-Even et al., 2006; Elowitz et al., 2002; Newman et al., 2006).

## Declaration of competing interest

The authors declare the following financial interests/personal relationships which may be considered as potential competing interests:

J.C.C. is a co-founder and equity holder in Prometheus Materials Inc. All other authors declare no competing interests.
