# Supplementary material for: Dynamic and single cell characterization of a CRISPR-interference toolset in Pseudomonas putida KT2440 for β-ketoadipate production from p-coumarate
Source: Metab Eng Commun. 2022 Aug 28;15:e00204. doi: 10.1016/j.mec.2022.e00204 (PMC9460563; doi:10.1016/j.mec.2022.e00204)
Supplement: Multimedia component 1 [file mmc1.pdf]

Supplementary Materials and Methods

Cloning

All PCR was conducted using New England BioLabs Inc. (NEB) Q5® High-fidelity DNA Polymerase 2x Master Mix using standard protocols. Primer annealing temperatures were calculated using NEB Tm Calculator (http://tmcalculator.neb.com/#!/main) with the Q5 High-fidelity DNA Polymerase kit and 500 nM primer concentration as calculator input parameters. Primers and synthetic genes were ordered from Eurofins Genomics as well as IDT. Amplified DNA was purified and concentrated using Qiagen® QIAquick PCR Purification Kit and any residual plasmid template was digested using DpnI (NEB). Digested product was gel purified using Qiagen® QIAquick Gel Extraction Kit and then dialyzed by spotting the gel purified DNA onto membrane filter (MF-Millipore™, 0.025 µm pore size) floating on ddH_2O. All vectors were assembled using the NEBuilder® HiFi DNA Assembly Kit. Assembled vectors were electroporated into either *E. coli* EPI400 (Lucigen®), *E. coli* 10G (Lucigen®), or *Pseudomonas putida* KT2440 wild type depending on the vector (see next paragraph for details). Colonies were isolated and inoculated into LB with appropriate antibiotic for plasmid extraction. Plasmids were extracted using the Qiagen Miniprep Kit and whole plasmids or PCR amplicons were Sanger sequenced using GeneWiz®.

dCas9 Integration vectors pK18sB::1K_d*cas9*_PP4785 and pK18sB::8K_d*cas9*_PP4785

The dCas9 integrations vectors introduce an insulated and inducible dCas9 expression cassette into a neutral site between the hypothetical genes PP_4785 and PP_4786 in the *P. putida* KT2440 chromosome. These were built with the pK18sB suicide vector backbone which uses the *kan/sacB* selection/counterselection cassette to create scar free edits (Jayakody et al. 2018). First, an intermediate integration vector containing A 1,200 bp region with the PP_4785/PP_4786 hypothetical genes as future homology arms was amplified from the *P. putida* KT2440 genome using primers F_PP4785/R_PP4785.

This was assembled with a linearized pK18sB amplified using primers F_pK18sB/R_pK18sB to make an intermediate plasmid pK18sB::PP_4785 containing the homology region PP_4758 for subcloning.

pK18sB::1K_d*cas9*_PP4785 and pK18sB::8K_d*cas9*_PP4785 were constructed with three piece Gibson assemblies. The1K-J23107 inducible promoter was amplified from plasmid pBBR1k-J23107-GFPuv with primers F_1K/R_1K_Cas9 and the 8K inducible promoter was amplified from pBb(B5)8k-sfGFP with primers F_8K_insulated/R_8K_cas9. *dcas9* was amplified from a sequence verified plasmid with primers F_dcas9_PPRBS/R_dcas9. A common backbone was amplified from the intermediate pK18sB::PP_4785 with primers F_PP4785_dCas9/ R_PP4785_term.These three respective amplicons were Gibson assembled to construct pK18sB::1K_d*cas9*_PP4785 and pK18sB::8K_d*cas9*_PP4785 and electroporated into *E. coli* EPI400™ (Lucigen®) cells to reduce the copy number of these plasmids. These vectors were initially transformed into *E. coli* 10G (Lucigen®) but the resulting colonies had variable sizes and variability growing up from plates in liquid culture. Transforming into EPI400™ cells alleviated these subculturing issues.

gRNA expression vectors and targeted gene locations

The parent CRISPRi gRNA expression vector pJFgRNA-NT was cloned by assembling a synthetically synthesized gRNA via IDTs gBlock and a linearized pBBR1-MSC2 backbone amplified with primers F_pBBR1_gRNA/R_pBBR1_gRNA. The N20 targeting region of the gRNA was swapped out with oligos. A forward and reverse oligo was designed that annealed at the N20 region and contained homology for Gibson assembly with the linearized backbone. The two oligos were PCR stitched to produce a double stranded DNA product. This was assembled with a linearized pJFgRNA-NT plasmid, amplified with primers Cre4/R_gRNA_JE1.

Primer stitching reaction conditions were as follows: 1 µL of each of the two 100 µM stock of oligos was added to a standard 20 µL Q5 PCR reaction. The reaction was cycled with an initial denaturation step of

98°C for 30 seconds, then fifteen cycles of 98°C for 10 seconds, the N20 spacer annealing temperature for 20 seconds, and a 72°C extension for 15 seconds, followed by a final extension step of 2 minutes. Then 1 μL of the PCR reaction was added to the assembly reaction without purification.

The locations of the genes targeted in this study are as follows: *ftsZ* [105,305 -> 106,456] (2.27 centisomes, 8°). *dnaA* [3,882,326 <- 3,883,729] (83.64 centisomes, 301°). *rpoD* [3,213,047 -> 3,214,888] (69.22 centisomes, 249°). pcaI [4,457,363 -> 4,458,058] (72.1 centisomes, 260°). *pcaJ* [4,458,067 -> 4,458,708] (72.12 centisomes, 260°).

sfGFP integration vectors

sfGFP was codon optimized by altering low frequency codons, defined here as below ten occurrences out of one thousand codons in the *P. putida* KT2440 chromosome, to their most frequent synonymous codon. The specific changes are as follows: GGA to GGC, CUU to CTG, CUU to CTG, UUA to CUG, GGG to GGC, ACA to ACC, CUA to CUG, AUA to AUC, UCA to UCG, and UAA to UGA.

Cloning the sfGFP integration vectors proved to be non-trivial. The high (JE111211) and low (JE131311) strength promoter/RBS pairs were chosen because they had been characterized in the genome of *P. putida* KT2440 (Elmore et al. 2018). As these promoters were derivatives of the tac promoter, their expression level in *E.* coli is high and expressing fluorescent proteins with these promoters is toxic. In order to streamline cloning in *E. coli*, the authors in the Elmore et al. 2018 study placed the 24 bp lactose operator (lacO) directly downstream of the transcription start site of the promoters and used *E. coli* 5-alpha F'Iq (NEB) as a cloning strain. This host overexpresses the lacIq repressor to reduce toxicity from protein overexpression. These promoters then behave as un-regulated constitutive promoters in *P. putida* because it lacks lacIq. For this study, the lac operator had to be removed so that the lacI protein expressed in the 1K-J23107 IPTG inducible promoter would not interfere with the sfGFP expression. Without the lacIq repression in *E. coli*, these constructs were impossible to clone and all transformations resulted in colonies with promoter mutations. Because *P. putida* KT2440 doesn't express these promoters as highly as *E. coli,* it was able to serve as a subcloning strain with no toxicity issues.

The pK18sB vector does not replicate in *P. putida*, and so a Cas9/λ-red editing system developed by Sun el al. 2018 was chosen to integrate sfGFP because it uses a *P. putida* compatible replicating plasmid and because all plasmids can be easily cured from the host after successful editing. First an intermediate editing plasmid, pSEVA-PP_2984-g2 was constructed from pSEVA-gRNAT with homology arms and gRNA designed to integrate sfGFP in between the inward facing hypothetical PP_2984 and PP_2985 genes. The PP_2984 amplicon was created with primers oJF1003/oJF1004 by amplifying from purified *P. putida* KT2440 genomic DNA. The pSEVA-gRic6T template was linearized with oJF1001/oJF1002, and the new gRNA design was synthesized as a gBlock™ from IDT named gRNA_PP2984_2. These two amplicons and gene product were Gibson assembled and transformed into *E. coli* 10G and sequence verified.

Next, high strength sfGFP cassettes were synthesized using IDTs gBlock™ Gene Fragment assembly service. These cassettes are insulated with intrinsic terminator sequences and contain a *P. putida* KT2440 codon optimized sfGFP gene expressed from the constitutive JE111211 (high expression) promoters with the lacO sequence removed (Elmore et al. 2018). The two synthetic sfGFP expression cassettes were PCR amplified with primers oJF1013/oJF1014 to add additional homology arm length for assembly. pSEVA-PP_2984-g2 was linearized with primers oJF1011/oJF1012 that were designed to omit a 29 bp region in the center of the PP_2984 homology arm that would serve as the targeting site for genomic gRNA cleavage. The assembly reaction was then electroporated into *P. putida* KT2440 and transformants were selected on Gent 35. Plasmids were miniprepped using the same Qiagen Miniprep Kit with the standard protocol including the optional PB wash step to remove any Dnase activity. Miniprep yields from *P. putida* KT2440 were lower than yields from the *E. coli* cloning strains. Agarose gels of the miniprep showed a distinct triplet band but with more smearing than a typical *E. coli* miniprep. These minipreps were used identically as those derived from traditional *E. coli* cloning strains.

Electroporation and Conjugation

P. puitda transformations were conducted with a room temperature protocol adapted from Choi et al. 2006 (Choi et al. 2006).Freezer stocks were inoculated into 25 mL flask cultures of LB with appropriate antibiotics and grown up overnight. In the morning, the cells were pelleted at 4,100xg for 10 minutes at room temperature, the supernatant was decanted, and then the cells were resuspended in the same volume of room temperature 10% glycerol. This wash step was repeated for two total washes and then pellet was resuspended in 1/100th of the initial culture volume of room temperature 10% glycerol. 50μL of the electrocompetent cells were mixed with either 500 ng – 1 μg plasmid DNA for suicide vectors or 50-100 ng plasmid DNA for all other vectors. The mixture was placed in a room temperature 0.1 cm gap electroporation cuvette. The cells were electroporated at 1600 V with time constants between 4-5 ms in an Eppendorf Eporator®. Immediately after electroporation cells were rescued with 1 mL of room temperature LB and placed in a 1.5 mL sterile microcentrifuge tube to incubate at 30°C, 200 RPM for one hour for simple transformations or two hours for suicide vector transformations. Dilutions were spread on LB agar with appropriate antibiotics. *E. coli* cloning strains were purchased electrocompetent and were electroporated according to the manufacturers protocols.

Conjugation of plasmids into *P. putida* KT2240 were mediated by *E. coli* S-17 cells. *E. coli* S-17 containing the plasmid to be conjugated and the *P. putida* KT2440 recipient were grown up from freezer stock overnight in LB with appropriate antibiotics. These two cultures were pelleted at 4,100xG for 10 minutes at room temperature, the supernatant was decanted, and the cells were resuspended in the same volume of 1X M9 salts. The OD_{600} of the washed cells was measured to be between 2-3 and the cells were mixed so that the OD_{600} ratio was 1:1. Then 25 μL of the mixture was spotted on LB agar plates, allowed to dry, and incubated at 30°C overnight. The grown cells were scraped off the plate and resuspended in LB and diluted onto LB agar plates containing appropriate antibiotic as well as 100 μg/mL carbenicillin and grown overnight at 30°C in order to select for single colonies of successful *P. putida* KT2440 conjugants. These colonies were picked and streaked again onto identical agar plates to remove any

residual *E. coli* S-17 cells. These purified colonies were then grown up in LB with appropriate antibiotic overnight and frozen for future use in experiments.

Strain construction

dCas9 integrated strains sJF1KdCas9 and sJF8KdCas9 were constructed by using the pK18sB kan/sacB selection counterselection method with suicide plasmids pJF1005 and pJF1006 which were designed to integrate the 1K and 8K dCas9 expression systems into the PP_4786 locus respectively (see cloning supplement). *P. putida* KT2440 cells were transformed with either pJF1005 or pJF1006 and the whole transformation was plated on LB agar + Kan50. Four to six colonies were re-streaked on LB agar+kan50 to further purify each integrant. To select for the removal of the Kan/SacB backbone, four to six colonies were then streaked on YT+25% sucrose plates. Eight colonies from the YT sucrose plates were again re-streaked on YT+25% sucrose plates to assure no carry over of cells that still contained the kan/sacB cassette. One colony from each of eight re-streaks was grown up in LB overnight and frozen. The colonies were tested for kanamycin sensitivity and the integrated dCas9 expression cassette was sequenced to assure successful integration.

Parent sfGFP integrated strains were constructed using the Cas9/λ-red editing pCAS-RK2K from Sun. et al. 2018 with *P. putida* KT2440 as the background strain. pCAS-RK2K was conjugated into *P. putida* KT2440 from *E. coli* S-17 (Sun et al 2018).Cas9/λ-red genome editing was conducted using a modified protocol from Sun et al 2018. sJF1031 was grown up overnight in LB+kan. In the morning, the λ-red operon was induced at a final concentration of 0.6% arabinose by adding 20% sterile filtered arabinose. The cultures were placed back in the incubator for 1 hour. Cells were made electrocompetent and transformed with 100 ng of either pJF1061. Dilutions were spread on LB + kan50+ gent10 plates and grown overnight at 30°C. Colonies were PCR amplified to verify successful sfGFP integration and colonies with correct band lengths were cured of pCAS-RK2K and pJF1061 by first growing up in LB + 10 mM rhamnose overnight. The overnight culture was streaked on LB agar + 10 g/L sucrose to cure

pCAS-RK2K. Colonies were tested for antibiotic sensitivity and the genomic sfGFP cassette was sequence verified. The 1K and 8K dCas9 systems were then integrated into this fluorescent strain using the above protocol to construct strains sJF1KdCas9F and sJF8KdCas9F.

Knockdown strains were constructed by transforming the desired gRNA plasmid into the appropriate dCas9 expression strain described above. Cells were plated on LB agar + Kan50 and single colonies were inoculated into LB+kan50 for overnight incubation and then frozen for use in experiments.

Table 1: Comprehensive Strain List

Paper Strain Name	Description	Source
P. putida KT2440	WT	ATCC® 47054
sJF1KdCas9	WT integrated with 1K dCas9	This work
sJF8KdCas9	WT integrated with arabinose inducible dCas9 (8K)	This work
sJF1KdCas9F	WT integrated with high sfGFP and with IPTG inducible dCas9 (1K)	This work
sJF8KdCas9F	WT integrated with high sfGFP and with arabinose inducible dCas9 (8K)	This work
sJF1KdCas9+gNT	sJF1KdCas9 transformed with non-targeting pJFgRNA_NT	This work
sJF8KdCas9+gNT	sJF8KdCas9 transformed with non-targeting pJFgRNA_NT	This work
sJF1KdCas9F+gNT	sJF1KdCas9F transformed with non-targeting pJFgRNA_NT	This work
sJF8KdCas9F+gNT	sJF8KdCas9F transformed with non-targeting pJFgRNA_NT	This work
sJF1KdCas9F+gGFP-C1	sJF1KdCas9F transformed with coding strand targeting pJFgRNA_GFP-C1	This work
sJF1KdCas9F+gGFP-C2	sJF1KdCas9F transformed with coding strand targeting pJFgRNA_GFP-C2	This work
sJF1KdCas9F+gGFP-C3	sJF1KdCas9F transformed with coding strand targeting pJFgRNA_GFP-C3	This work
sJF1KdCas9F+gGFP-C4	sJF1KdCas9F transformed with coding strand targeting pJFgRNA_GFP-C4	This work
sJF1KdCas9F+gGFP-T1	sJF1KdCas9F transformed with template strand targeting pJFgRNA_GFP-T1	This work
sJF1KdCas9F+gGFP-T2	sJF1KdCas9F transformed with template strand targeting pJFgRNA_GFP-T2	This work
sJF1KdCas9F+gGFP-T3	sJF1KdCas9F transformed with template strand targeting pJFgRNA_GFP-T3	This work
sJF1KdCas9F+gGFP-T4	sJF1KdCas9F transformed with template strand targeting pJFgRNA_GFP-T4	This work
sJF8KdCas9F+gGFP-C1	sJF8KdCas9F transformed with coding strand targeting pJFgRNA_GFP-C1	This work

sJF8KdCas9F+gGFP-C2	sJF8KdCas9F transformed with coding strand targeting pJFgRNA_GFP-C2	This work
sJF8KdCas9F+gGFP-C3	sJF8KdCas9F transformed with coding strand targeting pJFgRNA_GFP-C3	This work
sJF8KdCas9F+gGFP-C4	sJF8KdCas9F transformed with coding strand targeting pJFgRNA_GFP-C4	This work
sJF8KdCas9F+gGFP-T1	sJF8KdCas9F transformed with template strand targeting pJFgRNA_GFP-T1	This work
sJF8KdCas9F+gGFP-T2	sJF8KdCas9F transformed with template strand targeting pJFgRNA_GFP-T2	This work
sJF8KdCas9F+gGFP-T3	sJF8KdCas9F transformed with template strand targeting pJFgRNA_GFP-T3	This work
sJF8KdCas9F+gGFP-T4	sJF8KdCas9F transformed with template strand targeting pJFgRNA_GFP-T4	This work
sJF8KdCas9F+g*ftsZ*-1	sJF8KdCas9F transformed with coding strand targeting pJFgRNA_*ftsZ*-1	This work
sJF8KdCas9F+g*ftsZ*-2	sJF8KdCas9F transformed with coding strand targeting pJFgRNA_*ftsZ*-2	This work
sJF8KdCas9F+g*ftsZ*-3	sJF8KdCas9F transformed with coding strand targeting pJFgRNA_*ftsZ*-3	This work
sJF8KdCas9+g*rpoD*-1	sJF8KdCas9 transformed with coding strand targeting pJFgRNA_*rpoD*-1	This work
sJF8KdCas9+g*rpoD*-2	sJF8KdCas9 transformed with coding strand targeting pJFgRNA_*rpoD*-2	This work
sJF8KdCas9+g*rpoD*-3	sJF8KdCas9 transformed with coding strand targeting pJFgRNA_*rpoD*-3	This work
sJF8KdCas9+g*dnaA*-1	sJF8KdCas9 transformed with coding strand targeting pJFgRNA_*dnaA*-1	This work
sJF8KdCas9+g*dnaA*-2	sJF8KdCas9 transformed with coding strand targeting pJFgRNA_*dnaA*-2	This work
sJF8KdCas9+g*dnaA*-3	sJF8KdCas9 transformed with coding strand targeting pJFgRNA_*dnaA*-3	This work
sJF1KdCas9+g*pcaIJ*-1	sJF1KdCas9 transformed with coding strand targeting pJFgRNA_*pcaIJ*-1	This work
sJF1KdCas9+g*pcaIJ*-2	sJF1KdCas9 transformed with coding strand targeting pJFgRNA_*pcaIJ*-2	This work
sJF8KdCas9+g*pcaIJ*-1	sJF8KdCas9 transformed with coding strand targeting pJFgRNA_*pcaIJ*-1	This work
sJF8KdCas9+g*pcaIJ*-2	sJF8KdCas9 transformed with coding strand targeting pJFgRNA_*pcaIJ*-2	This work

Table 2: Additional Plasmids used in this work

Name	Description	Citation
pBBR1MCS-2	Broad host origin plasmid for gene expression	(Kovach et al. 1995) (Addgene Plasmid #85168)
pK18sB	Suicide vector with Kan selection and SacB counterselection	(Jayakody et al. 2018) (Genbank: MH166772)
pBBR1k-J23107-GFPuv	Contains 1K-J23107 IPTG inducible promoter	(Cook et al. 2018)

pBb(B5)8k-sfGFP	Contains 8K arabinose inducible promoter	(Cook et al. 2018)
pCAS-RK2K	Cas9/λ-red, sacB, plasmid with rhamnose inducible gRNA that targets pSEVA-gRic6T backbone for curing	(Sun et al. 2018)
pSEVA-gRic6T (pSEVA-gRNAT)	Cas9 Editing Cassette with gRNA expression vector that replicates in P. putida KT2440	(Sun et al. 2018)
pK18sB::1K_d*cas9*_PP4785	pK18sB sucicide vector with 1K-J23107 (IPTG) promoter system expressing dCas9 flanked by PP_4785 homology arms	This work
pK18sB::8K_d*cas9*_PP4785	pK18sB sucicide vector with 8K (arabinose) promoter system expressing dCas9 flanked by PP_4785 homology arms	This work
pSEVA_PP2984_gRNA#2_sfGFPhigh	pSEVA_PP2984_gRNA#2_sfGFPhigh	This work

Table 3: gRNA spacer sequences used in this work

gRNA expression Vector	Spacer Sequence	description
pJFgRNA-NT (gNT)	CAGCCACGAGATTTGAGGTA	Non-targeting gRNA Ptac expression vector with pBBR1MCS-2 ORI
pJFgRNA_GFP-C1 (gGFP-C1)	CATCCAGTTCAACCAGAATT	targets sfGFP at P13 (38 bp), coding strand
pJFgRNA_GFP-C2 (gGFP-C2)	CATCACCTTCACCCTCGCCA	targets sfGFP at R30 (89 bp), coding strand
pJFgRNA_GFP-C3 (gGFP-C3)	ATAGGTCAGAGTAGTGACCA	targets sfGFP at L60 (178 bp), coding strand
pJFgRNA_GFP-C4 (gGFP-C4)	GACTTCAGCACGCGTCTTGT	targets sfGFP at Y106 (316 bp), coding strand
pJFgRNA_GFP-T1 (gGFP-T1)	TGTCCCAATTCTGGTTGAAC	targets sfGFP at L18 (32 bp), template strand
pJFgRNA_GFP-T2 (gGFP-T2)	GGTGAAGGTGATGCTACCAA	targets sfGFP at N39 (96 bp), template strand
pJFgRNA_GFP-T3 (gGFP-T3)	TTGGTCACTACTCTGACCTA	targets sfGFP at Y66 (177 bp), template strand
pJFgRNA_GFP-T4 (gGFP-T4)	ACTATCTCTTTCAAAGATGA	targets sfGFP at D103 (288 bp), template strand
pJFgRNA_*ftsZ*-1 (gftsZ-1)	ACTTTAATGACCGGACTTTG	targets *ftsZ* at P9, coding strand

pJFgRNA_*ftsZ*-2 (gftsZ-2)	ACGCCGATCACTTTAATGAC	targets *ftsZ* at P12, coding strand
pJFgRNA_*ftsZ*-3 (gftsZ-3)	ACCCGTGCCCAATTGCAGGA	targets *ftsZ* at T57, coding strand
pJFgRNA_*rpoD*-1 (grpoD-1)	CATAACACCCTATCCACTGA	targets *rpoD* at M1, coding strand
pJFgRNA_*rpoD*-2 (grpoD-2)	CTGAAATATCCTCAGGCAGG	targets *rpoD* at H32, coding strand
pJFgRNA_*rpoD*-3 (grpoD-3)	GTCGGCTTCCGCCAACAGAA	targets *rpoD* at L69, coding strand
pJFgRNA_*dnaA*-1 (gdnaA-1)	TATCCCCTGAGTTTGAAAGC	targets *dnaA* at the RBS, coding strand
pJFgRNA_*dnaA*-2 (gdnaA-2)	CTGCCAAAGTTCCACTGACA	targets *dnaA* at V1, coding strand
pJFgRNA_*dnaA*-3 (gdnaA-3)	CGGCTTCGACCTGTAGCGGA	targets *dnaA* at R27, coding strand
pJFgRNA_*pcaIJ*-1 (gpcaIJ-1)	AGGCCATCGATCAGCTCGGA	targets *pcaIJ* at P33, coding strand
pJFgRNA_*pcaIJ*-2 (gpcaIJ-2)	GCTGCCTGCCATGAGCAGGG	targets *pcaIJ* at A63, coding strand

Table 4: Oligos used in this work

Oligo Name	Sequence 5'-3'	Used to construct
F_pK18sB	tcctctagagtcgacctgcaggc	pK18sB::1K_d*cas9*_PP4785, pK18sB::8K_d*cas9*_PP4785
R_pK18sB	gctcgaattcgtaatcatgtcatagctg	pK18sB::1K_d*cas9*_PP4785, pK18sB::8K_d*cas9*_PP4785
F_PP4785	TCACACAGGAAACAGCTATGACATGATTACGAATTCGAGCccataatgtgccaccagagacattgg	pK18sB::1K_d*cas9*_PP4785,

		pK18sB::8K_d*cas9*_PP4785
R_PP4786	CAGTGCCAAGCTTGCATGCCTGCAGGTCGACTCTAGAGGAgaagggttcgaggatgaaacgatgg	pK18sB::1K_d*cas9*_PP4785,<br><br>pK18sB::8K_d*cas9*_PP4785
F_PP4785_dCas9	CGCTCGGCCCGTGGACAACCCTGCTGGACAAGCTGATGGAggtcacttttccggcattggc	pK18sB::1K_d*cas9*_PP4785,<br><br>pK18sB::8K_d*cas9*_PP4785
R_PP4785_term	ATCATGATCTGATCAAGAGACAGGATGAGGATCGTTTCGCGGACCAAAACGAAAAAAGGCCCCCCTTTCGGGAGGCCTCTTTTCTGGAATTTGGTACCGAGttcagggaagcggcgagaac	pK18sB::8K_d*cas9*_PP4785
F_dcas9_PPRBS	AGCAGGACGCACTGACCAGGAGGTACAATCAatggataagaaatactcaataggctta	pK18sB::1K_d*cas9*_PP4785,<br><br>pK18sB::8K_d*cas9*_PP4785
R_dcas9	tccatcagcttgtccagc	pK18sB::1K_d*cas9*_PP4785,<br><br>pK18sB::8K_d*cas9*_PP4785
F_1K	gcgaaacgatcctcatcctgtctc	pK18sB::1K_d*cas9*_PP4785
R_1K_Cas9	CTTATCCATTGATTGTACCTCCTGGTCAGTGCGTCCTGCTaattgttatccgctcacaattccacaca	pK18sB::1K_d*cas9*_PP4785
F_8K_insulated	CGAAACGAATCCTCATCCTGTCTCTTGATCAGATCATGATTttactttgcagggcttcccaacc	pK18sB::8K_d*cas9*_PP4785
R_8K_cas9	CTTATCCATTGATTGTACCTCCTGGTCAGTGCGTCCTGCTatggagaaacagtagagagttgcgataaaaagcgt	pK18sB::8K_*dcas9*_PP4785
oJF1001	gaattcatggtgtcaattaattaaaggcatcaaataaaacgaaaggc	pSEVA_PP2984_gRNA#2_sfGFPhigh

oJF1002	caagcttgcggccgcgtc	pSEVA_PP2984_gRNA#2_sfGFPhigh
oJF1003	TCGCCAGGGTTTTCCCAGTCACGACGCGGCCGCAAGCTTGctatctggtcgacctgctgcgtc	pSEVA_PP2984_gRNA#2_sfGFPhigh
oJF1004	aacaaatacttccctaatcgacacctattgcgttagc	pSEVA_PP2984_gRNA#2_sfGFPhigh
oJF1011	AGTGtgtgcgcgtggagcaacc	pSEVA_PP2984_gRNA#2_sfGFPhigh
oJF1012	gaatttggtaccgagACACTCGTTATTTGTCATAAGCTCAatgagttcagcctggtgcgaattaagtagg	pSEVA_PP2984_gRNA#2_sfGFPhigh
oJF1013	TGAGCTTATGACAAATAACGAGTGTctc	pSEVA_PP2984_gRNA#2_sfGFPhigh
oJF1014	ttatttaaatatttatgcggttgctccacgcgcacaCACTGTGAGATGATTAAACAAGTACttttgttatc	pSEVA_PP2984_gRNA#2_sfGFPhigh
F_pBBR1_sgRNA	TGCACTCTTCAGCCGCATAGCAAGAATGTCAAccgacatggaagccatcacaaacg	pJFgRNA-NT
R_pBBR1_sgRNA	GTAATCATACCTGACCTCCATAGAATGATGTGATATTAtgaatccgttagcgaggtg	pJFgRNA-NT
Cre4	gttttagagctagaaatagcaagttaaaataaggctag	pJFgRNA-NT
R_gRNA_JE1	gatcacattatacgagccgatgattaattg	pJFgRNA-NT

Table 5: Oligos used to construct the gRNA spacers

Oligo	Sequence	Description
oJF1104	ACTATTGACAATTAATCATCGGCTCGTATAATGTGATCCATCCAGTTCAACCAGAATT	F oligo GFP-C1
oJF1105	ctagccttattttaacttgctatttctagctctaaaacAATTCTGGTTGAACTGGATG	R oligo GFP-C1
oJF1108	ACTATTGACAATTAATCATCGGCTCGTATAATGTGATCCATCACCTTCACCCTCGCCA	F oligo GFP-C2
oJF1109	ctagccttattttaacttgctatttctagctctaaaacTGGCGAGGGTGAAGGTGATG	R oligo GFP-C2

oJF1112	ACTATTGACAATTAATCATCGGCTCGTATAATGTGATCATAGGTCAGAGTAGTGACCA	F oligo GFP-C3
oJF1113	ctagccttattttaacttgctatttctagctctaaaacTGGTCACTACTCTGACCTAT	R oligo GFP-C3
oJF1118	ACTATTGACAATTAATCATCGGCTCGTATAATGTGATCGACTTCAGCACGCGTCTTGT	F oligo GFP-C4
oJF1119	ctagccttattttaacttgctatttctagctctaaaacACAAGACGCGTGCTGAAGTC	R oligo GFP-C4
oJF1106	ACTATTGACAATTAATCATCGGCTCGTATAATGTGATCTGTCCCAATTCTGGTTGAAC	F oligo GFP-T1
oJF1107	ctagccttattttaacttgctatttctagctctaaaacGTTCAACCAGAATTGGGACA	R oligo GFP-T1
oJF1110	ACTATTGACAATTAATCATCGGCTCGTATAATGTGATCGGTGAAGGTGATGCTACCAA	F oligo GFP-T2
oJF1111	ctagccttattttaacttgctatttctagctctaaaacTTGGTAGCATCACCTTCACC	R oligo GFP-T2
oJF1114	ACTATTGACAATTAATCATCGGCTCGTATAATGTGATCTTGGTCACTACTCTGACCTA	F oligo GFP-T3
oJF1115	ctagccttattttaacttgctatttctagctctaaaacTAGGTCAGAGTAGTGACCAA	R oligo GFP-T3
oJF1116	ACTATTGACAATTAATCATCGGCTCGTATAATGTGATCACTATCTCTTTCAAAGATGA	F oligo GFP-T4
oJF1117	ctagccttattttaacttgctatttctagctctaaaacTCATCTTTGAAAGAGATAGT	R oligo GFP-T4
F_ftsZ1_P9	actattgacaattaatcatcggctcgtataatgtgatcACTTTAATGACCGGACTTTG	F oligo ftsZ-1
R_ftsZ1_P9	ctagccttattttaacttgctatttctagctctaaaacCAAAGTCCGGTCATTAAAGT	R oligo ftsZ-1
F_ftsZ1_P12	ttgacagctagctcagtcctaggtataatgctagcACGCCGATCACTTTAATGAC	F oligo ftsZ-2
R_ftsZ1_P12	ctagccttattttaacttgctatttctagctctaaaacGTCATTAAAGTGATCGGCGT	R oligo ftsZ-2
F_ftsZ1_T57	ACTATTGACAATTAATCATCGGCTCGTATAATGTGATCACCCGTGCCCAATTGCAGGA	F oligo ftsZ-3
R_ftsZ1_T57	CTAGccttattttaacttgctatttctagctctaaaacTCCTGCAATTGGGCACGGGT	R oligo ftsZ-3
oJF1047	ctagccttattttaacttgctatttctagctctaaaactcagtggatagggtgttatg	R oligo rpoD-1
oJF1048	ACTATTGACAATTAATCATCGGCTCGTATAATGTGATCcataacaccctatccactga	F oligo rpoD-1
oJF1049	ctagccttattttaacttgctatttctagctctaaaaccctgcctgaggatatttcag	R oligo rpoD-2

oJF1050	ACTATTGACAATTAATCATCGGCTCGTATAATGTGATCctgaaatatcctcaggcagg	F oligo rpoD-2
oJF1051	ctagccttattttaacttgctatttctagctctaaaacttctgttggcggaagccgac	R oligo rpoD-3
oJF1052	ACTATTGACAATTAATCATCGGCTCGTATAATGTGATCgtcggcttccgccaacagaa	F oligo rpoD-3
F_dnaA1_RBS	ACTATTGACAATTAATCATCGGCTCGTATAATGTGATCtatcccctgagtttgaaagc	F oligo dnaA-1
R_dnaA_RBS	CTAGccttattttaacttgctatttctagctctaaaacgctttcaaactcaggggata	R oligo dnaA-1
F_dnaA1_V1	ACTATTGACAATTAATCATCGGCTCGTATAATGTGATCctgccaaagttccactgaca	F oligo dnaA-2
R_dnaA_V1	CTAGccttattttaacttgctatttctagctctaaaactgtcagtggaactttggcag	R oligo dnaA-2
F_dnaA1_R27	ACTATTGACAATTAATCATCGGCTCGTATAATGTGATCcggcttcgacctgtagcgga	F oligo dnaA-3
R_dnaA_R27	CTAGccttattttaacttgctatttctagctctaaaactccgctacaggtcgaagccg	R oligo dnaA-3
oJF1089	ACTATTGACAATTAATCATCGGCTCGTATAATGTGATCaggccatcgatcagctcgga	F oligo pcaIJ-1
oJF1090	CTAGccttattttaacttgctatttctagctctaaaactccgagctgatcgatggcct	R oligo pcaIJ-1
oJF1091	ACTATTGACAATTAATCATCGGCTCGTATAATGTGATCgctgcctgccatgagcaggg	F oligo pcaIJ-2
oJF1092	CTAGccttattttaacttgctatttctagctctaaaacccctgctcatggcaggcagc	R oligo pcaIJ-2

Table 6: gBlocks used in this work

Name	Sequence	Description
gRNA_PP2984_2	AGGGCTAACGCAATAGGTGTCGATTAGGGAAGTATTTGTTttgacagctagctcagtcctaggtataatgctagcGCGGTTGCTCCACGCGCACAgttttagagctagaaatagcaagttaaaataaggctagtccgttatcaacttgaaaaagtggcaccgagtcggtgcttttttttgaggagctcggtacccggggatGAATTCATGGTGTCAATTAATTAAAGGCATCAAATAAAAC	Contains J23119 expressing gRNA with seed #1 targeting deleted region of PP_2984
oJF1082 - GB	TGAGCTTATGACAAATAACGAGTGTctcggtaccaaattccagaaaagaggcctcccgaaaggggggccttttttcgttttggtccactagtagtctctatggaggtcaggtatgattactaTTGACAattaatcatcggctcgTTTAA	gBlock. insulated sfGFP (PP

	TgtgatcagacccttaagattaactcacacAGGAGATatcatatgagcaa aGGCgaagaaCTGttcactGGCgttgtcccaattCTGgttgaaCTGga tggtgatgttaatGGCcacaaattttctgtccgtGGCgagggtgaaggtga tgctACCaacGGCaaactcaccCTGaaatttatttgcactactGGTaaa CTGcctgttccgtggccaACCTTGgtcactactctgacctatggtgttcaat gcttttcccgttatccggatcacatgaaacggcatgactttttcaagagtgcca tgcccgaaggttatgtacaggaacgcactATCtctttcaaagatgacGGCa cctacaagacgcgtgctgaagtcaagtttgaaggtgataccTTGgttaatcg tatcgagttaaagggtattgattttaaagaagatGGTaacattCTGGGCc acaaaCTGgagtacaactttaacTCGcacaatgtatacatcacggcagac aaacaaaagaatGGCatcaaagctaacttcaaaattcgccacaacgttga agatggttccgttcaaCTGgcagaccattatcaacaaaatactccaattggc gatggccctgtcCTCCTGccagacaaccattacctgtcgACCcaatctgtc CTGtcgaaagatcccaacgaaaagcgtgaccacatggtcCTGTTGgagt ttgtaactgctgctGGTattACCcatggcatggatgagctctacaaaTGAt ctagagacgaacaataaggcctccctaacgggggcctttttttattgataac aaaaGTACTTGTTTAATCATCTCACAGTG	optimized) with high promoter JE111211
JE111111_gRNA_gBl[illegible]	TAATATCACATCATTCTATGGAGGTCAGGTATGATTACTAT TGACAATTAATCATCGGCTCGTATAATGTGATCAGACCCAG CCACGAGATTTGAGGTAgttttagagctagaaatagcaagttaaaat aaggctagtccgttatcaacttgaaaaagtggcaccgagtcggtgcttttttt GCACTCTTCAGCCGCATAGCAAGAATGTCAACCGACATGG AAGCCATCACAAACG	gBlock. JE111111 promoter with LacO expressing mNeonGreen P60 gRNA

Supplemental References

Choi, K.-H., Kumar, A. and Schweizer, H.P. 2006. A 10-min method for preparation of highly electrocompetent *Pseudomonas aeruginosa* cells: application for DNA fragment transfer between chromosomes and plasmid transformation. *Journal of Microbiological Methods* 64(3), pp. 391–397.

Cook, T.B., Rand, J.M., Nurani, W., Courtney, D.K., Liu, S.A. and Pfleger, B.F. 2018. Genetic tools for reliable gene expression and recombineering in Pseudomonas putida. *Journal of Industrial Microbiology & Biotechnology* 45(7), pp. 517–527.

Jayakody, L.N., Johnson, C.W., Whitham, J.M., Giannone, R.J., Black, B.A., Cleveland, N.S., Klingeman, D.M., Michener, W.E., Olstad, J.L., Vardon, D.R., Brown, R.C., Brown, S.D., Hettich, R.L., Guss, A.M. and Beckham, G.T. 2018. Thermochemical wastewater valorization *via* enhanced microbial toxicity tolerance. *Energy & Environmental Science* 11(6), pp. 1625–1638.

Kovach, M.E., Elzer, P.H., Hill, D.S., Robertson, G.T., Farris, M.A., Roop, R.M. and Peterson, K.M. 1995. Four new derivatives of the broad-host-range cloning vector pBBR1MCS, carrying different antibiotic-resistance cassettes. *Gene* 166(1), pp. 175–176.

Sun, J., Wang, Q., Jiang, Y., Wen, Z., Yang, L., Wu, J. and Yang, S. 2018. Genome editing and transcriptional repression in Pseudomonas putida KT2440 via the type II CRISPR system. *Microbial Cell Factories* 17(1), p. 41.

Supplementary Figures

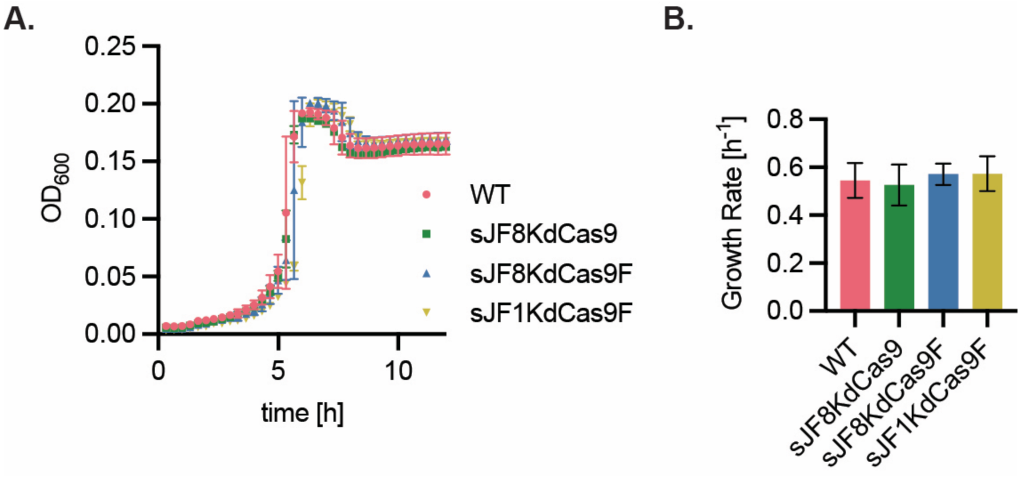

Figure S1: Growth profiles of dCas9 and sfGFP integrated strains in M9 + 30 mM glucose media. **A.** The dCas9 integrated base strains, sJF8KdCas9, sJF8KdCas9F, and sJF1KdCas9F were compared against the *P. putida* KT2440 WT strain in M9 + 30 mM glucose (no antibiotics), error bars represent the standard deviation of two biological replicates. **B.** Growth rates of strains in **A.** All strains had non-significant differences between the WT with a students *t*-test, (p>.05). All error bars represent the standard deviation between three biological replicates.

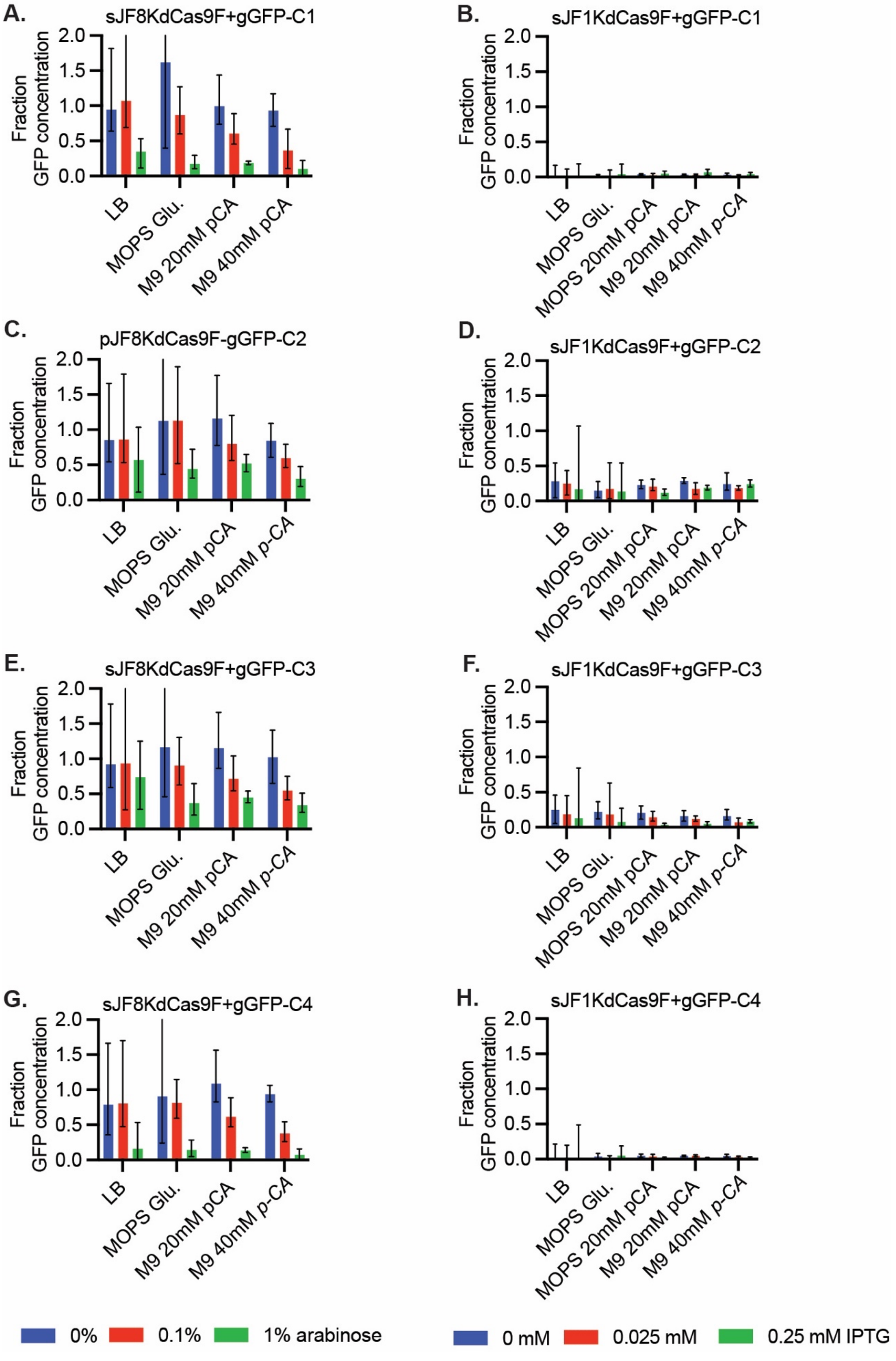

A.
sJF8KdCas9F+gGFP-C1
B.
sJF1KdCas9F+gGFP-C1
C.
pJF8KdCas9F-gGFP-C2
D.
sJF1KdCas9F+gGFP-C2
E.
sJF8KdCas9F+gGFP-C3
F.
sJF1KdCas9F+gGFP-C3
G.
sJF8KdCas9F+gGFP-C4
H.
sJF1KdCas9F+gGFP-C4
Fraction GFP concentration
2.0
1.5
1.0
0.5
0.0
LB
MOPS Glu.
MOPS 20mM pCA
M9 20mM pCA
M9 40mM pCA
M9 40mM p-CA
0%
0.1%
1% arabinose
0 mM
0.025 mM
0.25 mM IPTG

Figure S2: GFP titration with four sfGFP coding strand targeting guides in strain sJF8KdCas9F and sJF1KdCas9F. Plots show the fraction GFP concentration (same as GFP fraction) versus the +GFP control (sJF8KdCas9F+gNT or sJF1KdCas9F+gNT respectively) for a given strain gRNA pair versus media condition and induction concentration. **A. C. E.** and **G.** show strain sJF8KdCas9F+gGFP-C1, -C2, -C3, -C4 respectively in four media conditions with 0% (blue), 0.1% (red), and 1% (green) arabinose. **B. D. F**. and **H.** show strain sJF1KdCas9F+gGFP-C1, -C2, -C3, -C4 respectively in four media conditions with 0 (blue), 0.25mM (red), and 0.25mM (green) IPTG. Glu is 30 mM glucose, *p*-CA is p-coumaric acid. All conditions grown with Kan50. Error bars represent 95% confidence intervals calculated with triplicate biological replicates, see methods section 2.6. Values above 1 indicate that there was a higher GFP concentration in the experimental strain versus the positive GFP control strain, sJF8KdCas9F+gNT or sJF1KdCas9F+gNT.

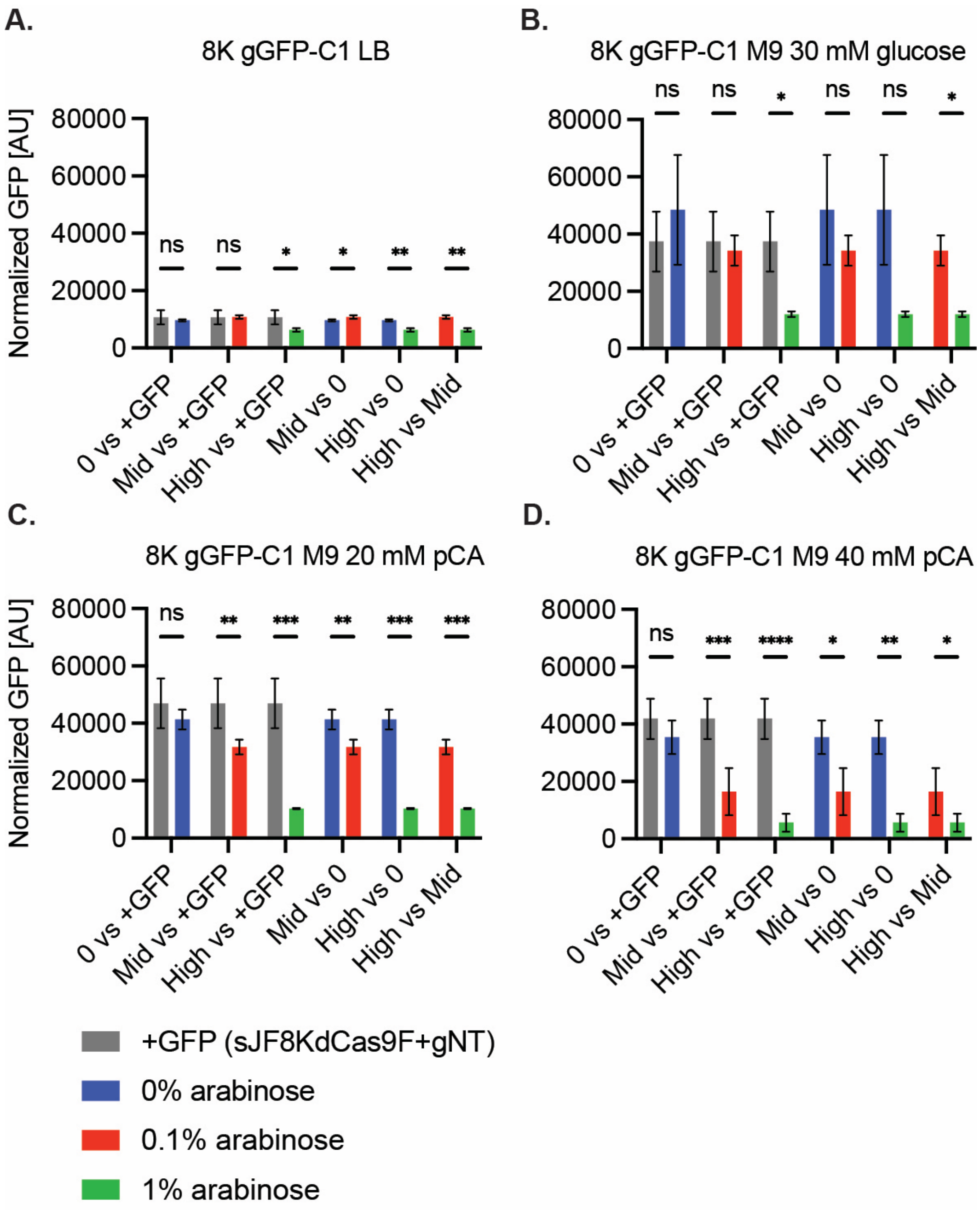

Figure S3: Statistical Analysis of GFP titration of strain sJF8KdCas9F+gGFP-C1 in various media conditions during exponential growth. Here normalized GFP [AU] is calculated as raw sfGFP fluorescence divided by OD_{600} at the given OD_{600} threshold in exponential phase (methods section 2.6). Each bar plot shows sJF8KdCas9F+gGFP-C1 grown in LB (**A.**), MOPS 30 mM glucose (**B.**), M9 20 mM pCA (**C.**), and M9 40 mM pCA (**D.**). No difference in normalized GFP was detected in the positive GFP control (+GFP, gray, sJF8KdCas9F+gNT) versus induction condition within a given media condition (one way ANOVA, $p>0.32$ or higher) and so data points were combined

from the three replicates in the three induction conditions for each media condition, n=9. The x axis legends are as follows: +GFP is the positive GFP control, sJF8KdCas9F+gNT, grey. 0 is 0% arabinose, blue. Mid is 0.1% (w/v) arabinose, red. High is 1% (w/v) arabinose, green. Bars represent the average between three biological replicates and error bars represent the standard deviation. Multiple unpaired t-tests were conducted between the groups of data shows with a false discovery rate of 5%. P values are noted above each grouped bar. Each asterisk, *, represents N digits after the p value decimal, ie. * is <0.05 and ** is <0.005. ns represents a non-significant difference given the false discovery rate chosen.

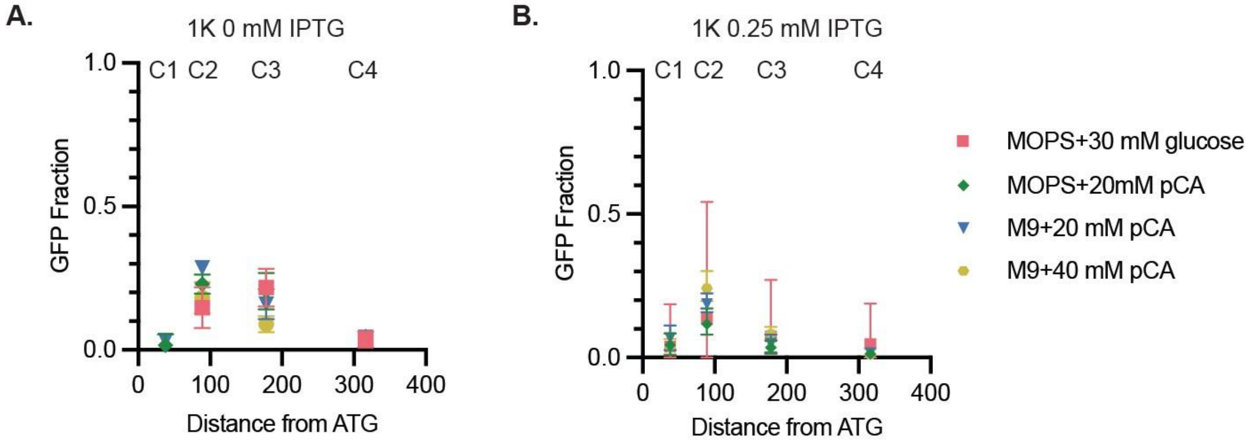

Figure S4: (**A-B**) GFP fraction for the 1K inducible promoter for 0, (**A.**)**,** and 0.25 mM IPTG (**B.**). **A.** & **B.** are plotted as a function of gRNA target location for 4 media conditions. gRNAs are named gGFP-C1, C2, C3, and C4 (labeled above axis); all four gRNAs target the strand encoding sfGFP. Error bars represent 95% confidence intervals of three biological replicates, see methods section 2.6.

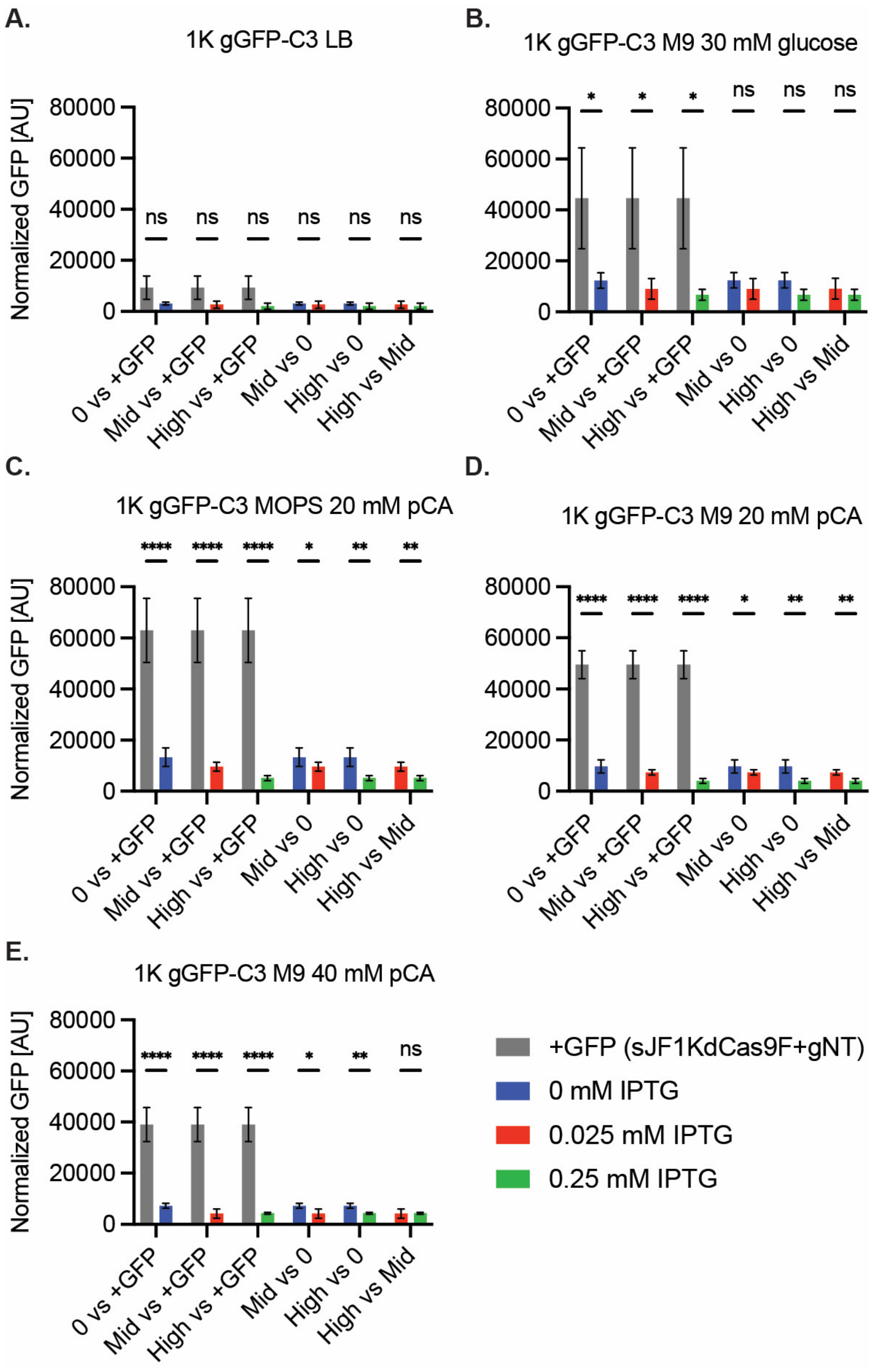
A.
1K gGFP-C3 LB
B.
1K gGFP-C3 M9 30 mM glucose
C.
1K gGFP-C3 MOPS 20 mM pCA
D.
1K gGFP-C3 M9 20 mM pCA
E.
1K gGFP-C3 M9 40 mM pCA
Normalized GFP [AU]
0
20000
40000
60000
80000
ns
*
**
****
0 vs +GFP
Mid vs +GFP
High vs +GFP
Mid vs 0
High vs 0
High vs Mid
+GFP (sJF1KdCas9F+gNT)
0 mM IPTG
0.025 mM IPTG
0.25 mM IPTG

Figure S5: Statistical Analysis of GFP titration of strain sJF1KdCas9F+gGFP-C3 in various media conditions during exponential growth. Here normalized GFP [AU] is calculated as raw sfGFP fluorescence divided by OD_{600} at the given OD_{600} threshold in exponential phase (methods section 2.6). Each bar plot shows sJF1KdCas9F+gGFP-C3 grown in LB (**A.**), MOPS 30 mM glucose (**B.**), M9 20 mM pCA (**C.**), and M9 40 mM pCA (**D.**). No difference in normalized GFP was detected in the positive GFP control (+GFP, gray, sJF1KdCas9F+gNT) versus induction condition within a given media condition (one way ANOVA, $p>0.32$ or higher) and so data points were combined from the three replicates in the three induction conditions for each media condition, n=9. The x axis legends are as follows: +GFP is the positive GFP control, sJF8KdCas9F+gNT, grey. 0 is 0 mM IPTG, blue. Mid is 0.025 mM IPTG, red. High is 0.25 mM IPTG, green. Bars represent the average between three biological replicates and error bars represent the standard deviation. Multiple unpaired t-tests were conducted between the groups of data shows with a false discovery rate of 5%. P values are noted above each grouped bar. Each asterisk, *, represents N digits after the p value decimal, ie. * is <0.05 and ** is <0.005. ns represents a non-significant difference given the false discovery rate chosen.

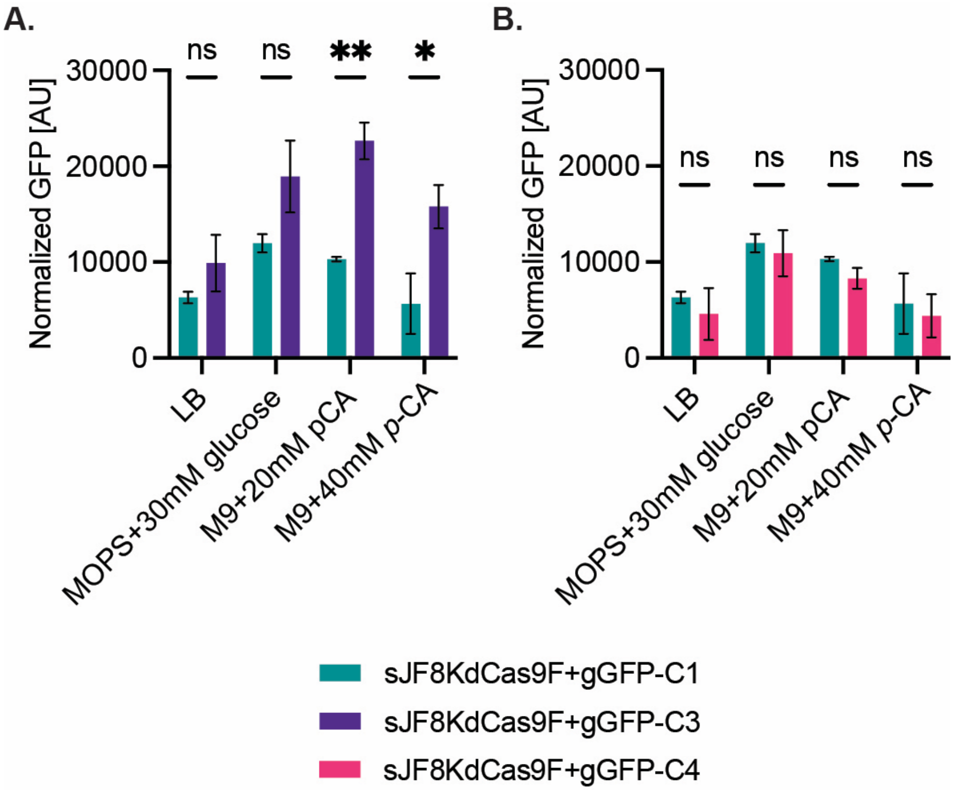

Figure S6: Statistical analysis comparing GFP knockdown of gGFP-C1, -C2, and -C3 in the 8K strain with 1% (w/v) arabinose in various media conditions. . Here normalized GFP [AU] is calculated as raw sfGFP fluorescence divided by OD_{600} at the given OD_{600} threshold in exponential phase (methods section 2.6) **A.** sJF8KdCas9F+gGFP-C1, teal, is compared to sJF8KdCas9F-C3, purple, in various media conditions all with 1% (w/v) arabinose. **B.** sJF8KdCas9F+gGFP-C1, teal, is compared to sJF8KdCas9F-C4, pink, in various media conditions all with 1% (w/v) arabinose. Bars represent the average between three biological replicates and error bars represent the standard deviation. Multiple unpaired t-tests were conducted between the groups of data shows with a false discovery rate of 5%. P values are noted above each grouped bar. Each asterisk, *, represents N digits after the p value decimal, ie. * is <0.05 and ** is <0.005. ns represents a non-significant difference given the false discovery rate chosen.

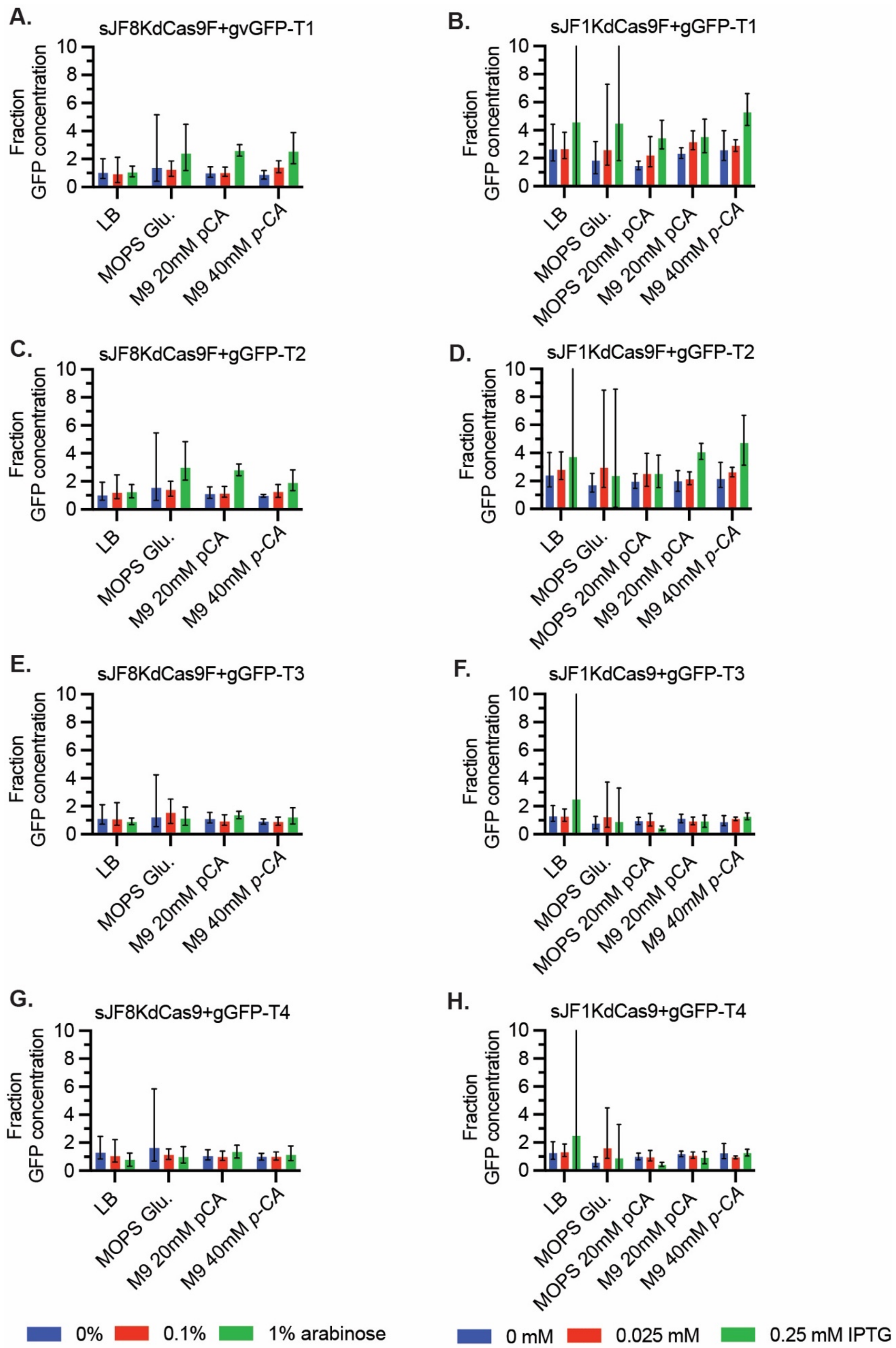
A.
sJF8KdCas9F+gvGFP-T1
B.
sJF1KdCas9F+gGFP-T1
C.
sJF8KdCas9F+gGFP-T2
D.
sJF1KdCas9F+gGFP-T2
E.
sJF8KdCas9F+gGFP-T3
F.
sJF1KdCas9+gGFP-T3
G.
sJF8KdCas9+gGFP-T4
H.
sJF1KdCas9+gGFP-T4
Fraction GFP concentration
0
2
4
6
8
10
LB
MOPS Glu.
MOPS 20mM pCA
M9 20mM pCA
M9 40mM p-CA
0%
0.1%
1% arabinose
0 mM
0.025 mM
0.25 mM IPTG

Figure S7: GFP titration with four sfGFP template strand targeting guides in strain sJF8KdCas9F and sJF1KdCas9F. Plots show the Fraction GFP concentration for a given gRNA strain pair versus media condition and percentage induction. A) C) E) and G) show strain sJF8KdCas9+gGFP-T1, -T2, -T3, -T4 respectively in four media conditions with 0% (blue), 0.1% (red), and 1% (green) arabinose. B) D) F) and H) show strain sJF8KdCas9+gGFP-T1, -T2, -T3, -T4 respectively in four media conditions with 0 (blue), 0.25mM (red), and 0.25mM (green) IPTG. Glu is 30 mM glucose, pCA is *p*-coumaric acid. All conditions grown with Kan50. Error bars represent 95% confidence intervals, see methods section 2.6. Values above 1 indicate that there was a higher GFP concentration in the experimental strain versus the positive GFP control strain, sJF8KdCas9F+gNT or sJF1KdCas9F+gNT.

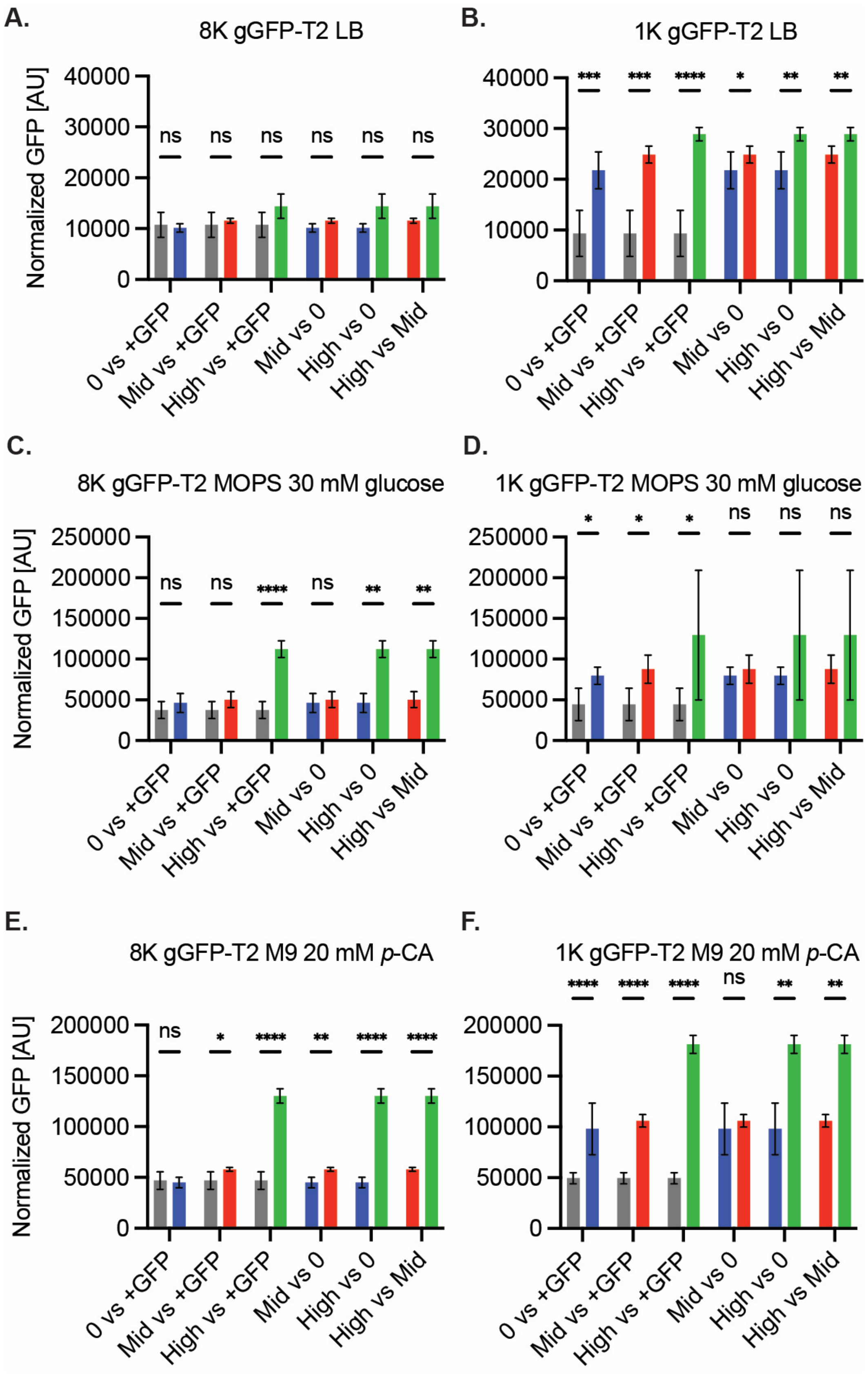

A.
8K gGFP-T2 LB
B.
1K gGFP-T2 LB
C.
8K gGFP-T2 MOPS 30 mM glucose
D.
1K gGFP-T2 MOPS 30 mM glucose
E.
8K gGFP-T2 M9 20 mM p-CA
F.
1K gGFP-T2 M9 20 mM p-CA
Normalized GFP [AU]
0 vs +GFP
Mid vs +GFP
High vs +GFP
Mid vs 0
High vs 0
High vs Mid
ns
*
**
***
****

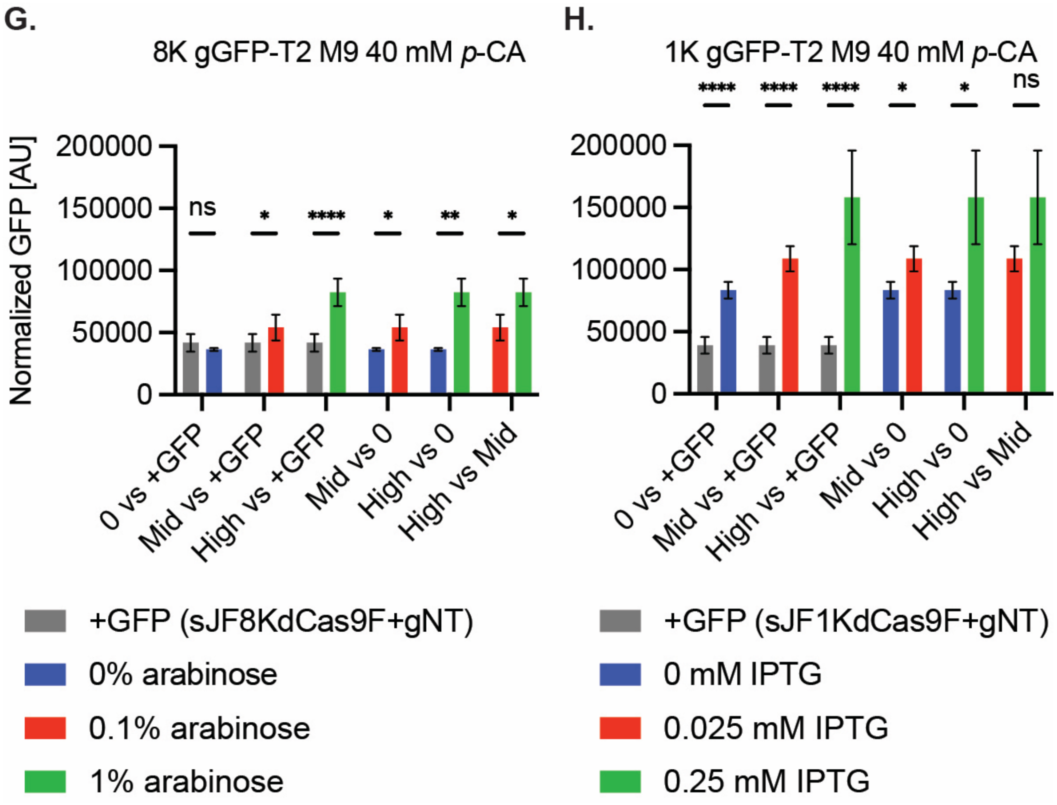

Figure S8: Statistical Analysis of GFP titration of strain sJF8KdCas9F+gGFP-T2 and sJF1KdCas9F+gGFP-T2 in various media conditions during exponential growth. Here normalized GFP [AU] is calculated as raw sfGFP fluorescence divided by OD_{600} at the given OD_{600} threshold in exponential phase (methods section 2.6). Left column of plots show sJF8KdCas9F+gGFP-T2 and right column of plots show sJF1KdCas9F+gGFP-T2 in LB (**A.** and **B.**), MOPS 30 mM glucose (**C.** and **D.**), M9 20 mM *p*-CA (**E.** and **F.**), and M9 20 mM *p*-CA (**G.** and **H.**). No difference in normalized GFP was detected in the positive GFP control (+GFP, gray, sJF8KdCas9+gNT or sJF1KdCas9F+gNT, respectively) versus induction condition within a given media condition (one way ANOVA, $p>0.32$ or higher) and so data points were combined from the three replicates in the three induction conditions for each media condition, n=9. The x axis legends are as follows: +GFP is the positive GFP control, sJF8KdCas9F+gNT or sJF1KdCas9F+gNT, respectively, grey. 0 is 0% arabinose or 0 mM IPTG, respectively, blue. Mid is 0.1% (w/v) arabinose or 0.025 mM IPTG, respectively, red. High is 1% (w/v) arabinose or 0.25 mM IPTG, respectively, green. Bars represent the average between three biological replicates and error bars represent the standard deviation. Multiple unpaired t-tests were conducted between the groups of data shows with a false discovery rate of 5%. P values are noted above each grouped bar. Each asterisk, *, represents N digits after the p

value decimal, ie. * is <0.05 and ** is <0.005. ns represents a non-significant difference given the false discovery rate chosen.

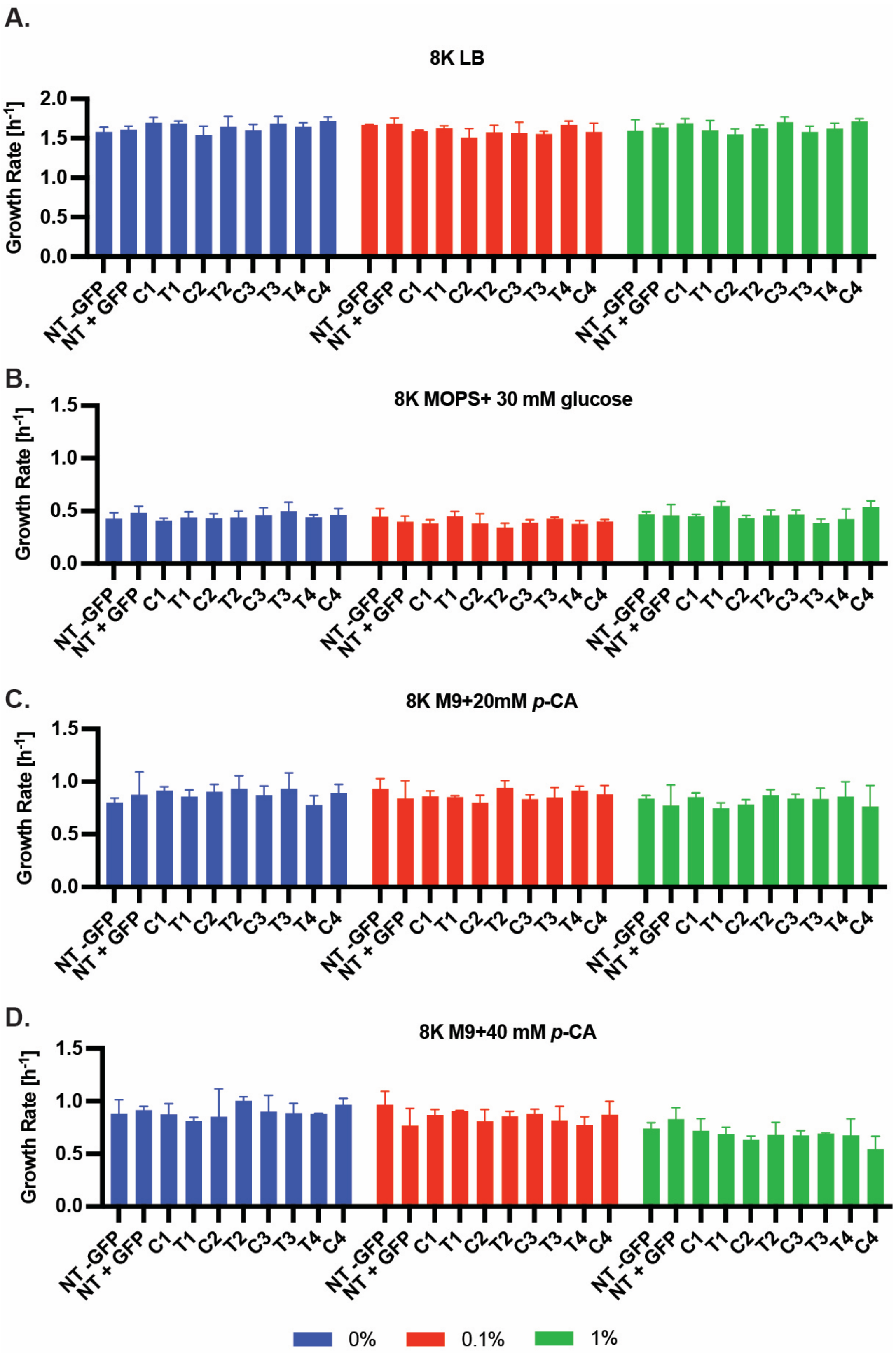
A.
8K LB
Growth Rate [h-1]
2.0
1.5
1.0
0.5
0.0
NT -GFP
NT + GFP
C1
T1
C2
T2
C3
T3
T4
C4
B.
8K MOPS+ 30 mM glucose
C.
8K M9+20mM p-CA
D.
8K M9+40 mM p-CA
0%
0.1%
1%

Figure S9: Growth rate of sFJ8KdCas9F versus media condition and induction. Plots show the growth rate vs sfGFP gRNA for sJF8KdCas9F in **A.** LB **B.** MOPS+30mM glucose **C.** M9+20 mM p-coumaric acid and **D.** M9+40 mM p-coumaric acid. Each media condition is grown with 0% (blue), 0.1% (red), or 1% (green) arabinose. NT -GFP is sJF8KdCas9+gNT, NT +GFP is sJF8KdCas9F+gNT, C1 is sJF8KdCas9F+gGFP-C1 etc. Students t test was conducted between the NT + GFP control in each media/induction condition versus each targeting gRNA. * represents a p value < 0.05. Error bars represent the standard deviation of three biological replicates.

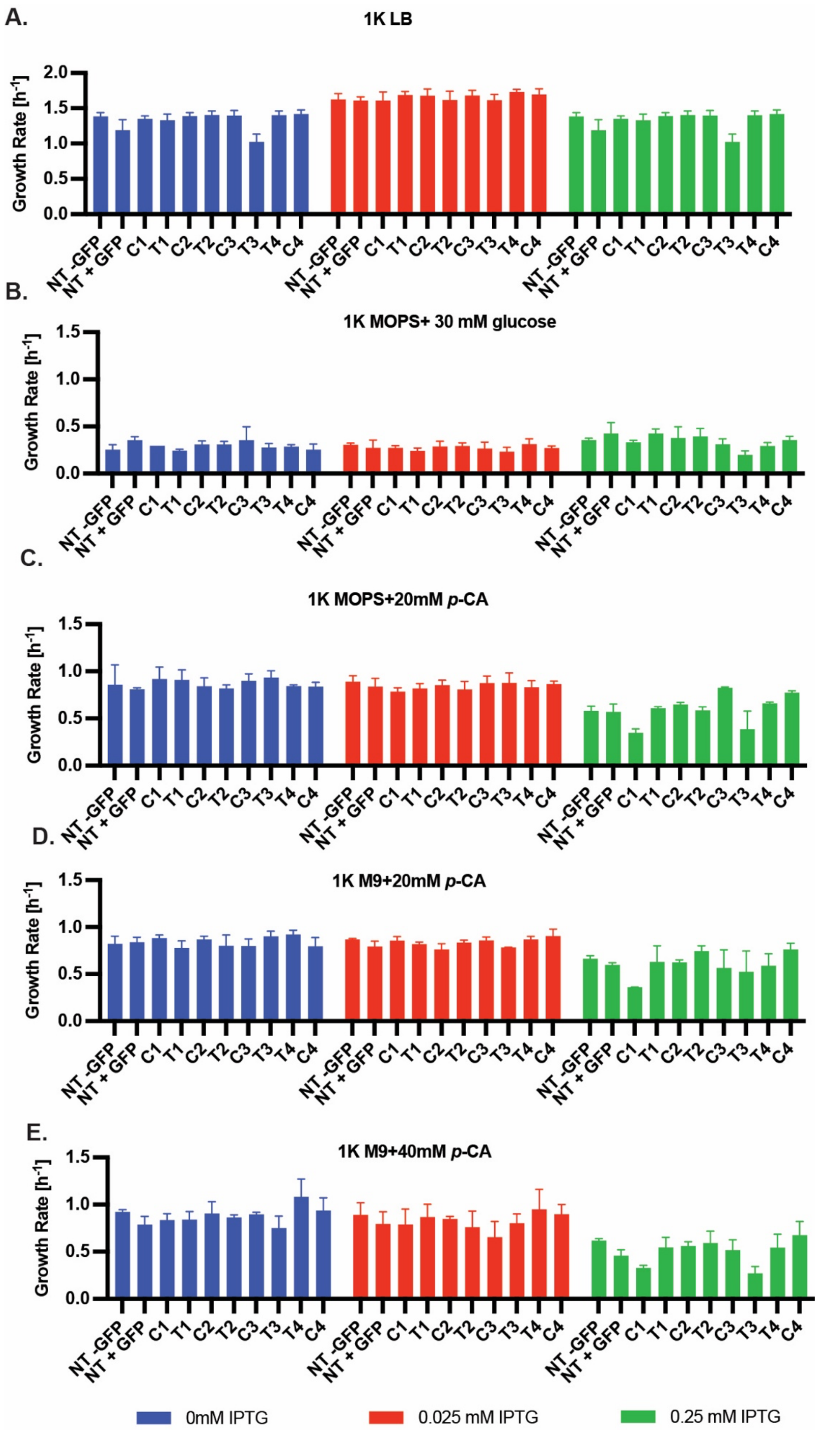

A.
1K LB
Growth Rate [h-1]
2.0
1.5
1.0
0.5
0.0
NT -GFP
NT + GFP
C1
T1
C2
T2
C3
T3
T4
C4
B.
1K MOPS+ 30 mM glucose
Growth Rate [h-1]
1.5
1.0
0.5
0.0
C.
1K MOPS+20mM p-CA
Growth Rate [h-1]
D.
1K M9+20mM p-CA
Growth Rate [h-1]
E.
1K M9+40mM p-CA
Growth Rate [h-1]
0mM IPTG
0.025 mM IPTG
0.25 mM IPTG

Figure S10: Growth rate of sFJ8KdCas9F versus media condition and induction. Plots show the growth rate vs sfGFP gRNA for sJF8KdCas9F in A) LB B) MOPS+30mM glucose C) M9+20 mM p-coumaric acid and D) M9+40 mM p-coumaric acid. Each media condition is grown with 0% (blue), 0.1% (red), or 1% (green) arabinose. NT -GFP is sJF8KdCas9+gNT, NT +GFP is sJF8KdCas9F+gNT, C1 is sJF8KdCas9F+gGFP-C1 etc. Students t test was conducted between the NT + GFP control in each media/induction condition versus each targeting gRNA. * represents a p value < 0.05. Error bars represent the standard deviation between three biological replicates.

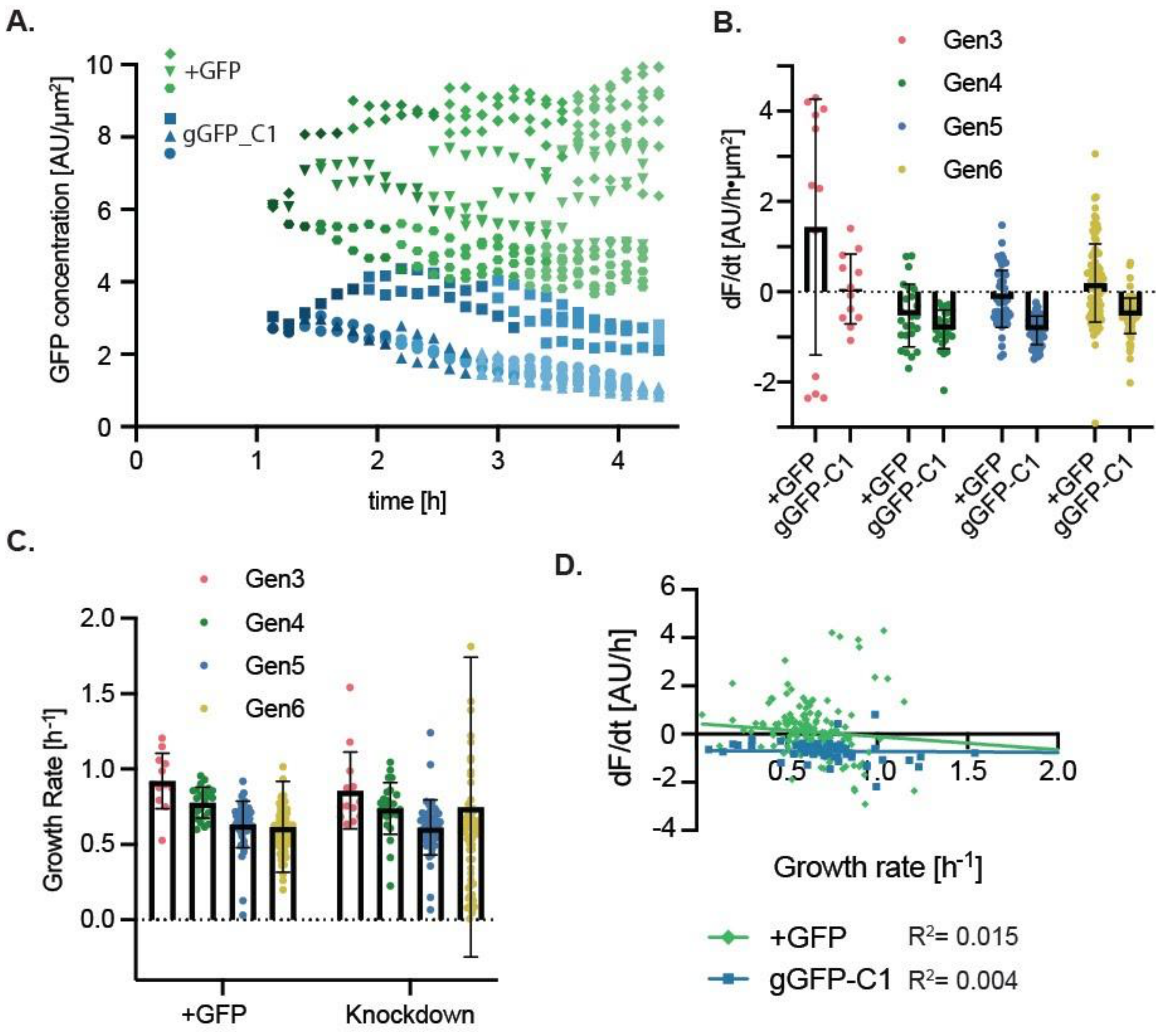

Figure S11: Single cell GFP knockdown additional data. A) The GFP concentration of individual cells over time as determined by single cell time lapse microscopy is plotted for the lineage of three +GFP cells (sJF8KdCas9F+gNT), green, and three GFP knockdown cells (sJF8KdCas9+gGFP-C1), blue, grown on M9+30mM glucose pads with 1% arabinose. Filming commenced ~1 hour after spotting cells during which two doublings had occurred (each micro-colony contained four cells at start of movie). Each plotted initial cell goes through three doublings, generations 3-6. Each lineage has its own shaped marker and the markers of each consecutive generation becomes lighter. B) The rate of change of GFP concentration over a cells lifespan, dF/dt (see methods section 2.9), is shown versus generation for the +GFP and knockdown strains (sJF8KdCas9F+gNT and sJF8KdCas9+gGFP-C1). Each circle represents the value of dF/dt across a cells lifespan. The bar plot shows the mean and the error bars represent the

standard deviation of all dF/dt values within the given condition C) Growth rates of sJF8KdCas9F+gNT and sJF8KdCas9F+gGFP-C1 cells by generation. Each circle represents the growth rate of an individual cell during its lifetime and the bars and whiskers represent the mean and standard deviation of growth rates respectively. Each circle represents the growth rate across a cells lifespan. The bar plot shows the mean and the error bars represent the standard deviation of all growth rates within the given condition D) The dF/dt vs growth rate is plotted for each cell in the +GFP (sJF8KdCas9F+gNT) and gGFP-C1 (sJF8KdCas9+gGFP-C1). Linear regression lines are fitted to the data and the R^2 values of each regression line are shown in the legend.

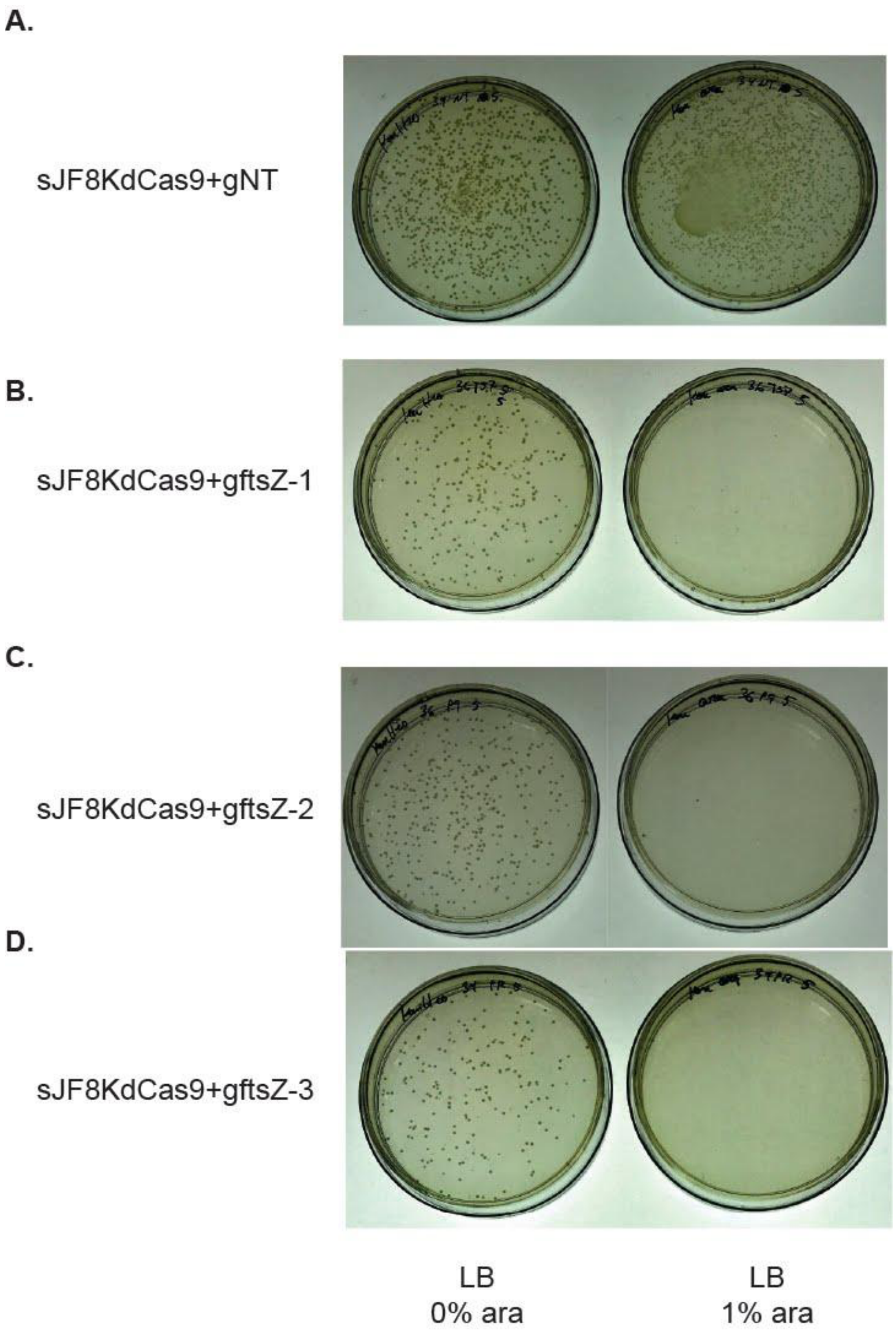

Figure S12: Guides targeting ftsZ were transformed into sJF8KdCas9 and 5 μL of the recovery medium was plated on LB kan50 with and without 1% arabinose. A) sJF8KdCas9 transformed with pNT B) C) and D) are sJF8KdCas9 transformed with g*ftsZ*-1, -2, and -3 respectively.

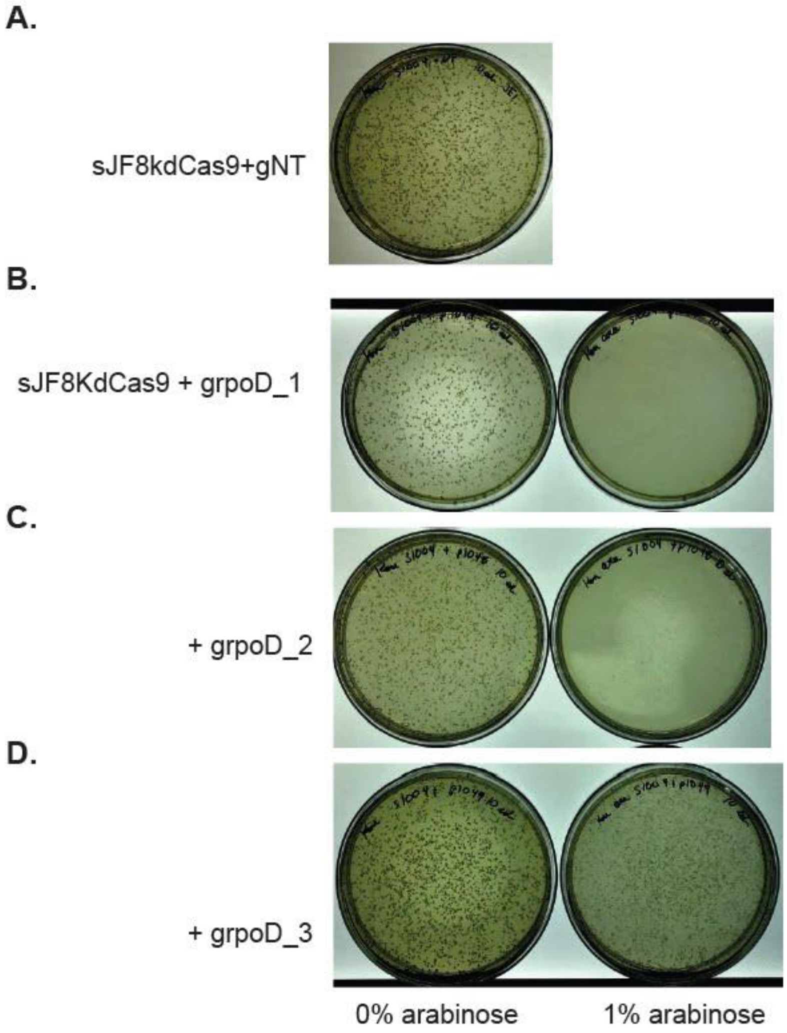

Figure S13: Guides targeting *rpoD* were transformed into sJF8KdCas9 and 10 μL of the recovery medium was plated on LB kan50 with and without 1% arabinose. A) sJF8KdCas9 transformed with pNT B) C) and D) are sJF8KdCas9 transformed with g*rpoD*-1, -2, and -3 respectively.

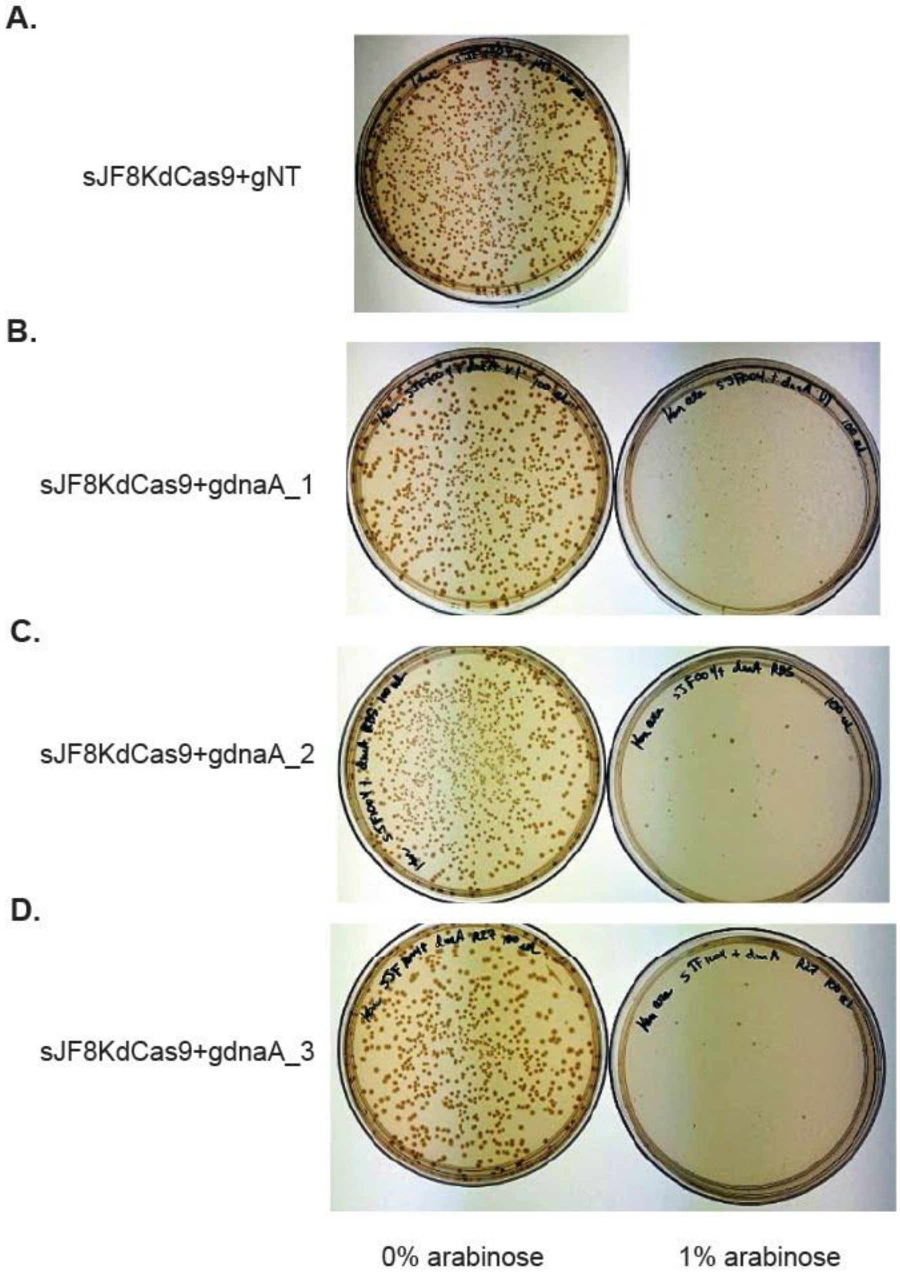

Figure S14: Guides targeting *dnaA* were transformed into sJF8KdCas9 and 10 μL of the recovery medium was plated on LB kan50 with and without 1% arabinose. A) sJF8KdCas9 transformed with pNT B) C) and D) are sJF8KdCas9 transformed with g*dnaA*-1, -2, and -3 respectively.

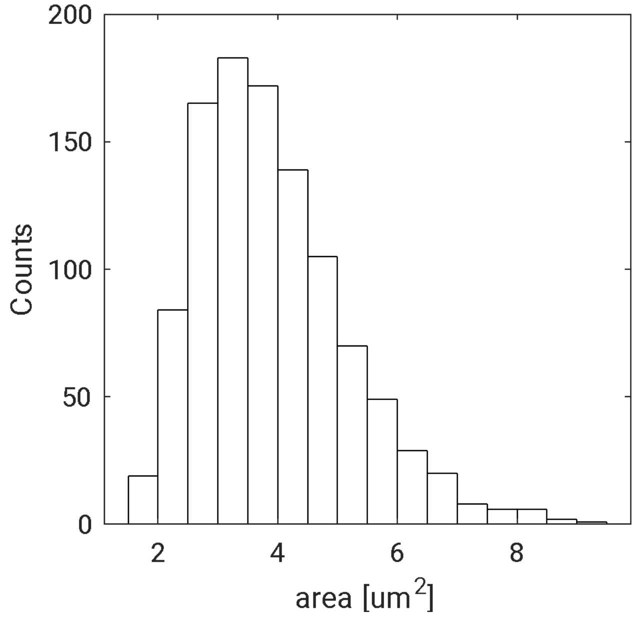

Figure S15: Area distribution of sJF8KdCas9+gNT cells. Cell areas from both LB and LB+1% arabinose growth conditions were combined on this histogram. 9 µm^2 was chosen as the threshold for a filamenting cell type because it is larger than the largest cell measured.

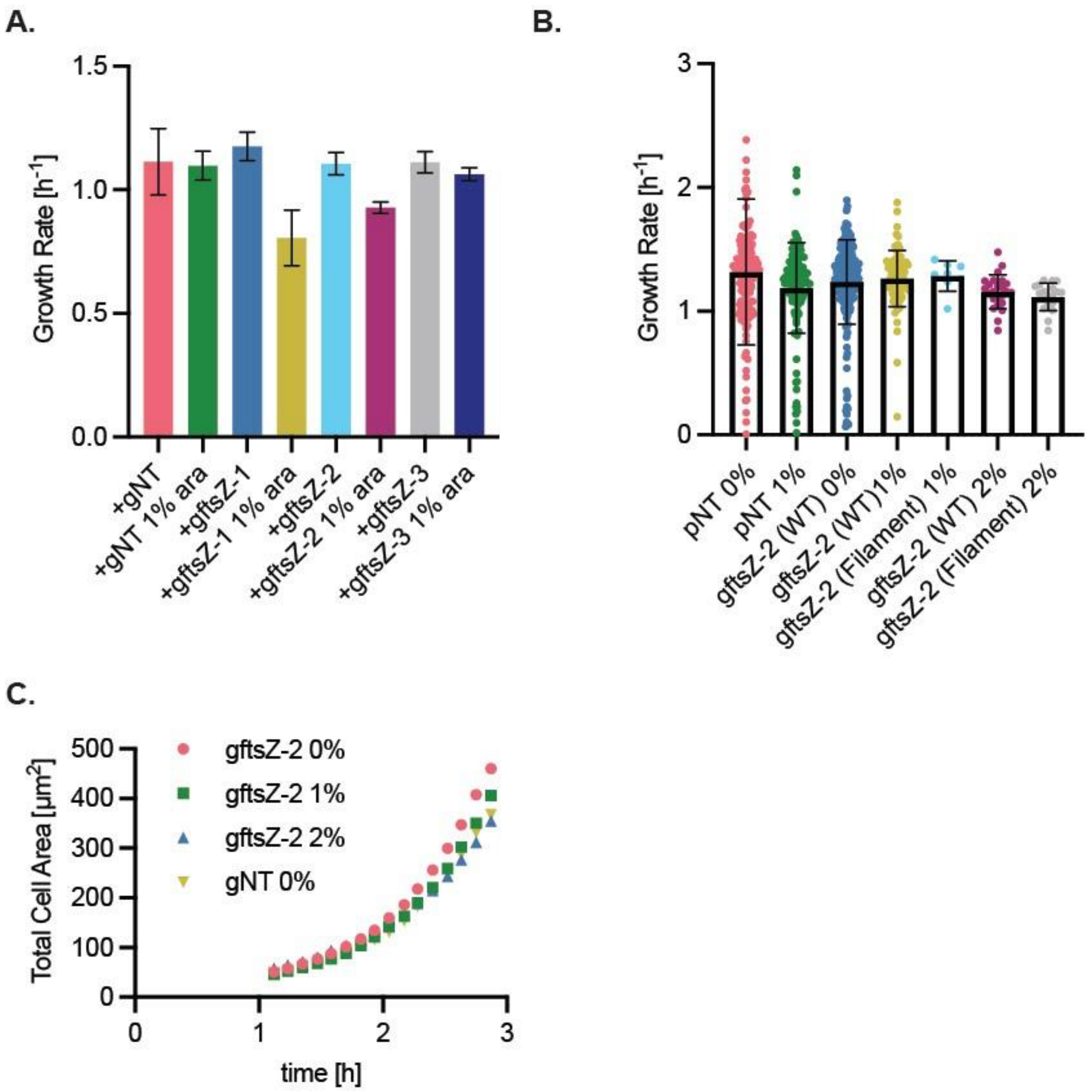

Figure S16: Bulk and Single cell *ftsZ* knockdown data of sJF8KdCas9+gNT or +gftsZ-2 grown on LB kan50 with various arabinose percentages. A) Bulk plate reader growth rates for sJF8KdCas9 transformed with the plasmids in the axis labels. Cells were grown in LB Kan50 +/- 1% arabinose (w/v) B) Single cell growth rate plotted versus strain, phenotype, and induction condition. WT refers to cells that divided like the wild type control (sJF8KdCas9+gNT) and filament refers to cells that reached an area above 9 μm^2 and never divided again. No filamenting cells were observed in the sJF8KdCas9+g*ftsZ*-2 0% arabinose condition. There was no difference in growth rate between the filamenting cells and NT cells (p value = 0.41). Each dot represents the growth rate of a cell through its lifetime. The bar plots mark the mean and error is the standard deviation of all the growth rates of the cells within the given condition C) the cumulative total area of segmented cells on each frame starting from the same

number of original cells in each condition. D) The cell count per generation for 0, 1, and 2% arabinose. Cells that filament never produce decedents that enter the next generation. Compare the number of cells in each generation to the total area of cells in B).

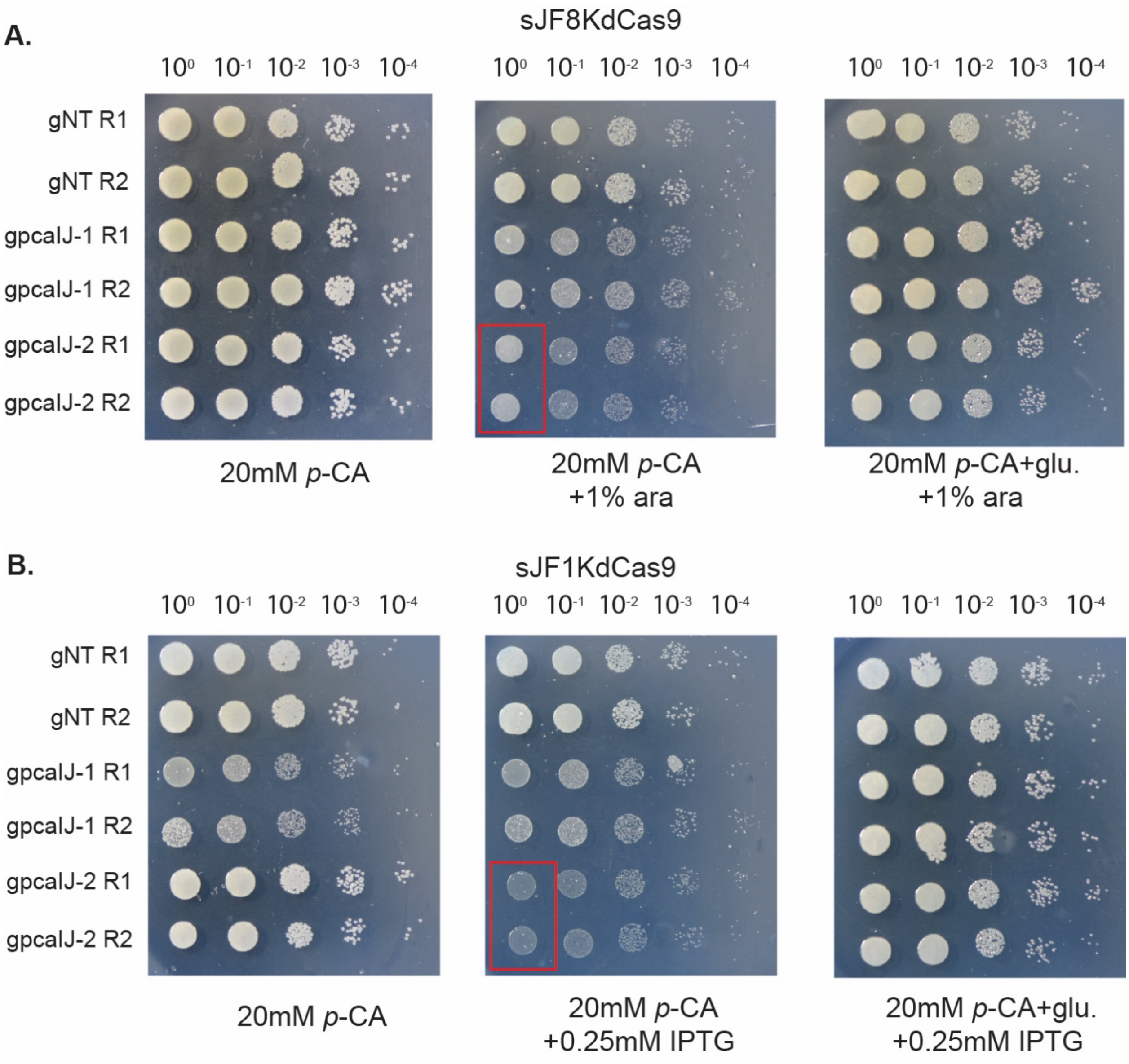

Figure S17: Spot plates of *pcaIJ* knockdown strains. A) sJF8KdCas9+gNT, +g*pcaIJ*-1, and +p*caIJ*-2 with two biological replicates, R1 and R2, were spotted at an OD_{600}=0.1 with four 10-fold dilutions on (left to right) M9+20mM pCA 1.5%, + 1% arabinose, and + 1% arabinose + 30 mM glucose (glu) agar pads. Images were taken 24 hours after start of growth. B) sJF1KdCas9+gNT, +g*pcaIJ*-1, and +*pcaIJ*-2 with two biological replicates, R1 and R2, were spotted at an OD_{600}=0.1 with four 10-fold dilutions on (left to right) M9+20mM pCA 1.5%, + 0.25 mM IPTG, and + 0.25mM IPTG + 30 mM glucose (glu) agar pads. Images were taken 24 hours after start of growth. Red boxes highlight the increased growth reduction seen with g*pcaIJ*-2 when expressed in sJF1KdCas9 vs sJF8KdCas9.

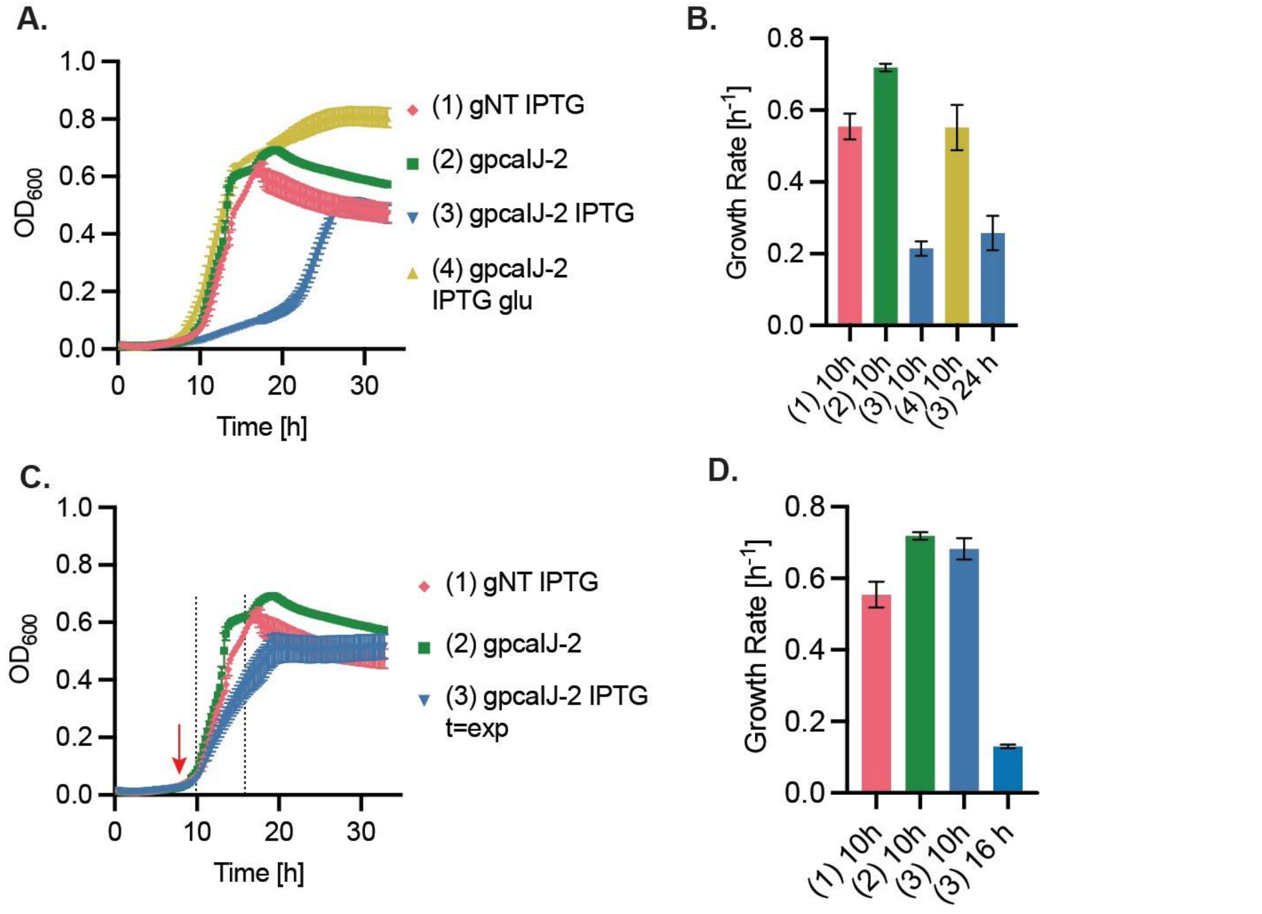

Figure S18: A) Growth curve of *pcaIJ* knockdown strain, sJF1KdCas9+g*pcaIJ*-2 (gpcaIJ-2) in MOPS+20mM *p*-CA. Only the induced knockdown strain, (3) g*pcaIJ*-2 IPTG, shows a growth defect. Removing IPTG, (2) g*pcaIJ*-2, or adding glucose, (4) g*pcaIJ*-2 IPTG glu, rescue growth to wild type levels, (1) gNT IPTG. **B.** Growth rates of cultures (1)-(4) from **A.** at the designated time point, 10 or 24 hours. **C.** Growth curve of *pcaIJ* knockdown strain induced during early exponential phase. The non-targeting control strain (1) and the uninduced knockdown strain (2) are re-plotted from **A.** for comparison. The *pcaIJ* knockdown strain, (3) g*pcaIJ*-2 IPTG t=exp, was induced at 9 hours, red arrow, during early exponential phase. **D.** Growth rates of cultures (1)-(3) from **C.** at the designated time point. All error bars represent the standard deviation between three biological replicates.

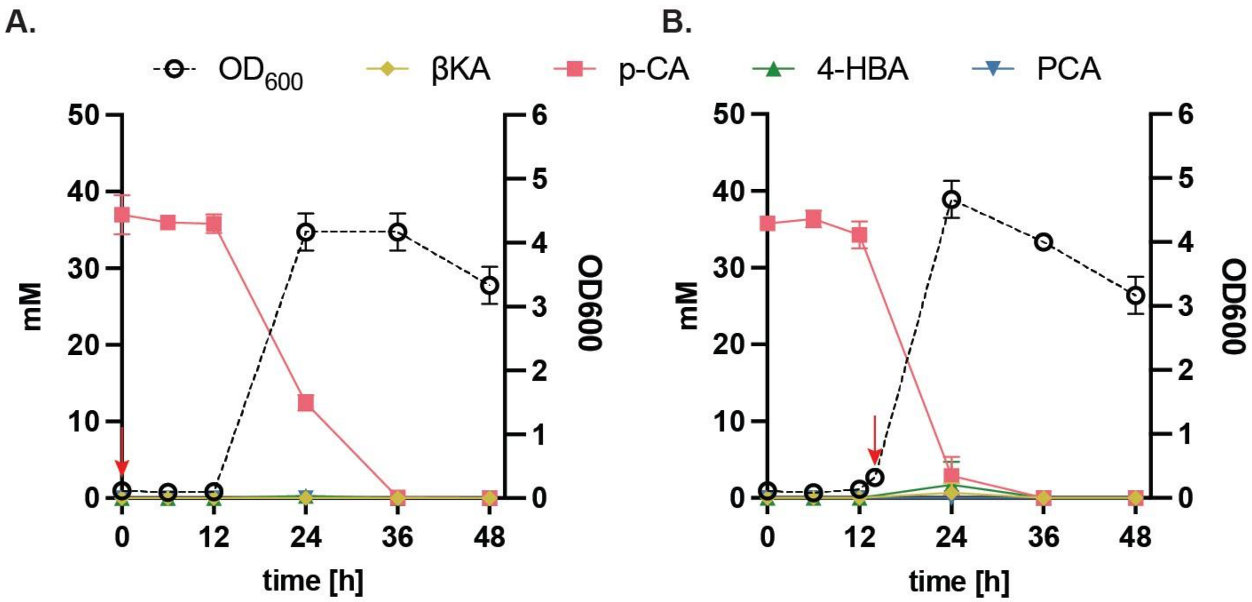

Figure S19: sJF1KdCas9+gNT fermentations in 40 mM p-CA. 50 mL cultures containing 40 mM p-CA were inoculated at OD_{600} = 0.1 with sJF1KdCas9+gNT and induced with 0.25mM IPTG at either time zero, **A.**, or 14 hours, **B.**, red arrow. The concentration of p-coumarate (p-CA), 4-hydroxybenzoate (4-HBA), protocatechuate (PCA), and β-ketoadipate (βKA), in the supernatant is plotted versus time alongside the OD_{600}. All error bars represent the standard deviation between three biological replicates.

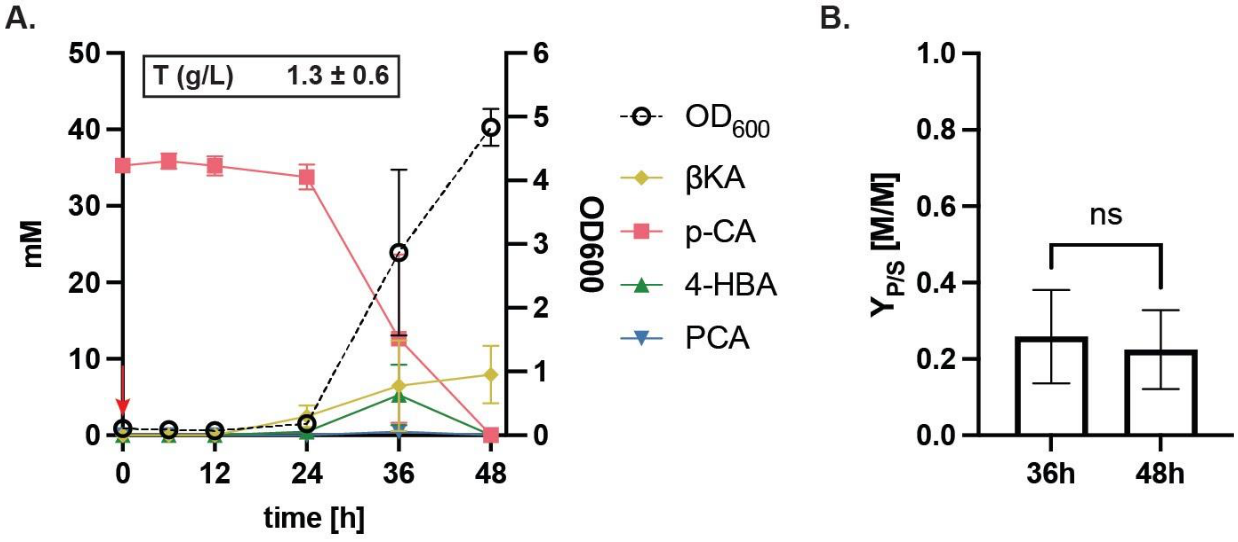

Figure S20: sJF1KdCas9+g*pcaIJ*-2 fermentations time zero inoculation in 40 mM p-CA. **A.** 50 mL cultures containing 40 mM p-CA were inoculated at OD_{600} = 0.1 with sJF1KdCas9+g*pcaIJ*-2 and induced with 0.25mM IPTG at time zero, red arrow. The concentration of p-coumarate (p-CA), 4-hydroxybenzoate (4-HBA), protocatechuate (PCA), and β-ketoadipate (βKA), in the supernatant is plotted versus time alongside the OD_{600}. Titer, T, molar yield, Y, were calculated at 48h when p-CA dropped to zero **B.** The product yield $Y_{P/S}$ is plotted for the 24h and 36h time point versus time zero. A paired t test between the 24 and 36h time point showed no significant difference (p=0.29). All error bars represent the standard deviation between three biological replicates.

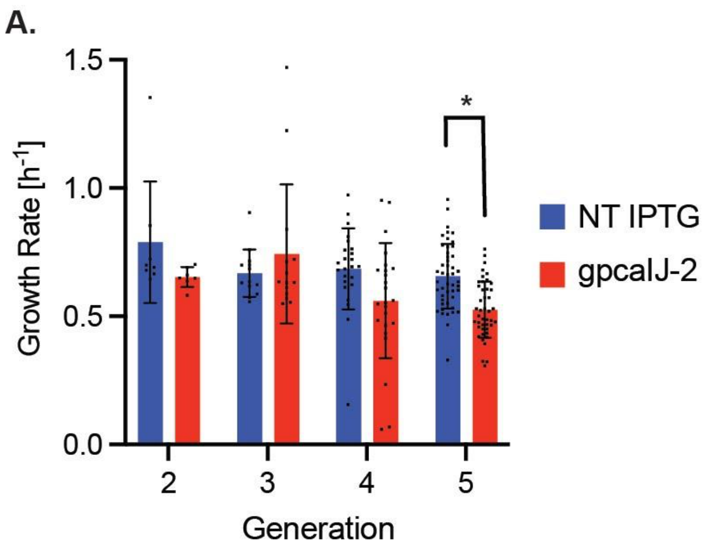

Figure S21: Single cell growth rate data grouped by generation for *pcaIJ* knockdown strain sJF1KdCas9+g*pcaIJ*-2 versus the non-targeting control sJF1KdCas9+gNT grown on 1.5% agar MOPS pads with 40mM *p*-CA as the sole carbon source and 0.25mM IPTG. It took four doublings on solid media to observe a reduced growth rate. * indicates a p value <0.05 on a students t test. The bar plots the mean growth rate with error bars representing the standard deviation of growth rates in that generation. Black dots represent measured growth rates of individual cells in that generation. Three colonies with two cells each in frame 1 were segmented through four hours of growth.
